# Characterization of Neuronal Ensembles in a Model of Dual-Reward Conditioned Place Preference

**DOI:** 10.1523/ENEURO.0463-25.2026

**Published:** 2026-06-24

**Authors:** Levi T. Flom, Kathryn L. Sandum, Skylar L. Hodgins, Samuel Johnson Noya, Jenna Crouse, Zhaojie Zhang, Ana-Clara Bobadilla

**Affiliations:** ^1^Department of Biomedical Sciences, Colorado State University, Fort Collins, Colorado; ^2^School of Pharmacy, University of Wyoming, Laramie, Wyoming; ^3^Department of Zoology and Physiology, University of Wyoming, Laramie, Wyoming

**Keywords:** c-Fos-TRAP2, chocolate, cocaine, dual-reward, neuronal ensembles, nucleus accumbens core, prefrontal cortex

## Abstract

Substance use disorder is characterized by maladaptive patterns of reward-seeking behavior. Such behavior has been linked to neuronal ensembles, typically identified through activity-dependent expression. These ensembles have been studied in regions such as the prelimbic prefrontal cortex and the nucleus accumbens core. In this study, we characterized ensembles tagged during concurrent exposure to cocaine- and chocolate-associated contexts. We used 33 male and 33 female Ai14xFos^2A-iCreERT2^ (c-Fos-TRAP2) transgenic mice to tag neuronal ensembles in a dual cocaine–chocolate conditioned place preference (CPP) paradigm, in which each chamber was associated with a different reward, either cocaine or chocolate. We found that after successful dual conditioning and in the absence of rewards, mice preferred the cocaine-paired chamber to the chocolate-paired chamber. Additionally, in mice exposed to both cocaine and chocolate, cortical and accumbal ensembles (measured as tdTomato^+^ cell density) tagged during the test session were comparable in size to those in single-reward conditions. However, TRAP2 neurons labeled during exposure to reward-paired contexts were larger than those in home cage control mice. We also found that the TRAP2-labeled ensemble did not correlate with the degree of chamber preference in either single- or dual-CPP models. These results introduce a dual-reward paradigm for studying context- and activity-dependent neuronal ensembles, in contrast to natural rewards used in noncontingent behavioral models.

## Significance Statement

Drug-related behaviors are linked to neuronal ensembles brain-wide, but less is known about drug-related characteristics of activity-tagged populations in the context of polyreward exposure. We developed a new noncontingent dual cocaine–chocolate conditioned place preference model that enabled us to compare how competition between activity-dependent cocaine and chocolate context determines tdTomato expression in the nucleus accumbens core and the prelimbic prefrontal cortex. These findings reveal that mice develop a preference for the cocaine-paired chamber over the chocolate-paired chamber when the reward is absent and characterize cocaine-context–tagged ensembles in animals that experienced a competing reward. Thus, our findings offer valuable insights into the neuronal differences between drug- and natural-reward ensembles.

## Introduction

Substance use disorder (SUD) is characterized by uncontrolled substance use despite adverse consequences, often leading to failures in social, financial, and personal responsibilities (Hasin et al., 2013). In this disorder, drug rewards are often prioritized over natural rewards, such as food. Both overlapping and specific neural circuitry and behavioral outcomes drive the seeking of natural and drug rewards (Nall et al., 2021). To examine changes underlying drug seeking, we crossed Ai14 x Fos^2A-iCreERT2^ (c-Fos-TRAP2) transgenic mice (DeNardo et al., 2019) to tag neuronal ensembles active during exposure to reward-associated contexts. c-*fos* is an immediate early gene commonly used as a marker to tag ensembles of cells during behavioral events (Bal et al., 2019). Extensive work has characterized neuronal ensembles across behavioral domains, including fear conditioning and SUD (Ramirez et al., 2013; Tonegawa et al., 2015; Bobadilla et al., 2017, 2020; Chen et al., 2019; Sieburg et al., 2019; Warren et al., 2019; Zhou et al., 2019; Kane et al., 2021; Roy et al., 2022; Hughes et al., 2024). Activity-defined neuronal ensembles have been used to study maladaptive behaviors associated with SUD, such as compulsive drug seeking, persistence despite adverse consequences, and increased relapse vulnerability, behaviors not observed in response to natural rewards (Baik, 2013). Throughout this study, we use the term neuronal ensemble to refer to TRAP2-labeled neurons expressing tdTomato (tdTomato^+^), reflecting activity-dependent tagging rather than causally linked ensembles.

While studies of single-drug exposure in animals have provided invaluable knowledge on the neurobiology of the reward circuitry, humans with SUD often engage with multiple drugs alongside natural reward sources (Mahoney et al., 2021). Polyreward studies examine combined rewards, involving either two pharmacological agents (polysubstance use) or a drug contrasted with natural rewards, such as sugar or social interaction. Preclinical rodent research on polysubstance SUD is typically conducted in rats, comparing two drugs of abuse as rewards (Crummy et al., 2020 ; Stennett and Knackstedt, 2020 ; Amico et al., 2022). Noncontingent studies of concurrent drug and natural rewards have similarly used rats, with socialization as the natural reward (Fritz et al., 2011; Zernig et al., 2013; Bregolin et al., 2017). Follow-up studies showed that PKA and CaMKII inhibition increase cocaine seeking in concurrent noncontingent studies while leaving socialization unaffected (Amaral et al., 2020, 2021). Similar work in mice (Kummer et al., 2014) estimated cocaine's relative reward strength to be 300-fold higher than in rats, underscoring species differences and suggesting that, unlike rats, mice exhibit weaker motivation for social contact when competing with drug rewards. These studies have yielded valuable insights. However, because social interaction and ingestible rewards differ fundamentally and because minimal work has examined ensemble-level neural characteristics associated with these reward types (Prast et al., 2014), we aimed to expand research on both polyreward exposure and the characteristics of activity-tagged ensembles. In this study, we investigated responses to cocaine and chocolate rewards in mice. Because previous work has primarily examined polyreward exposure and ensemble specificity in contingent self-administration (SA) paradigms (Cameron and Carelli, 2012; Bobadilla et al., 2020; Kane et al., 2021), we chose a noncontingent model to isolate context-dependent tagging from operant components of reward acquisition. Contingent models, such as SA, emphasize motivational and decision-making processes, as animals must perform an operant response to obtain the reward, which can confound the interpretation of neural activity related to reward exposure. In contrast, noncontingent paradigms, such as conditioned place preference (CPP), deliver rewards independently of the animal's behavior, allowing us to isolate ensemble-level responses to cocaine and chocolate without the influence of effort or choice. This distinction is critical for understanding how polyreward exposure influences activity patterns within relevant circuits beyond the strategies required to access them, complementing insights from contingent models (Green and Bardo, 2020).

Here, we present a noncontingent dual model in which each chamber is paired with a distinct reward of cocaine or chocolate allowing mice to build context associations for both rewards while enabling us to tag and quantify neuronal ensembles active during exposure to cocaine- or chocolate-associated contexts in the nucleus accumbens core (NAcore) and the prelimbic region (PL) of the prefrontal cortex, two regions that play a significant role in cocaine consumption and seeking (Thomas et al., 2008; James et al., 2018; Boggess et al., 2021; Kietzman et al., 2022). We also assessed tdTomato^+^ cell density in traditional single-reward CPP and examined correlations between tdTomato^+^ cell density and chamber preference.

## Materials and Methods

### Animals

All animal procedures were performed in accordance with the University of Wyoming and Colorado State University IACUC regulations. Male and female mice (7–24 weeks old) were generated by crossing female Ai14 knock-in mice [B6;129S6-Gt (ROSA)26Sortm14(CAG-tdTomato)Hze/J, stock #007914, RRID: IMSR_JAX:007914, the Jackson Laboratory] with c-Fos-TRAP2 male knock-in mice [STOCK Fostm2.1(icre/ERT2)Luo/J, stock #030323, RRID: IMSR_JAX:030323, the Jackson Laboratory]. Mice were individually housed; this housing condition does not induce stress or anxiety (Smolensky et al., 2024). Mice were placed on a reverse 12 h light cycle 3 d before the start of the experiment. All experiments were run during ZT 12–15. Mice in chocolate or dual-CPP experiments had 3 h daily access to food beginning on the preconditioning day. The 3 h window for food access was immediately after the conditioning session, with most of the time without food during the sleep cycle. This approach was used to increase the likelihood that mice consumed chocolate during conditioning to develop a place preference. Mice involved in single-cocaine CPP were given *ad libitum* food access. Mice involved in chocolate and dual cocaine–chocolate CPP experiments were given ten miniature chocolate chips (1 g Nestlé) in their home cage the day before preconditioning started to prevent neophobia. Mice were handled and received at least three vehicle injections intraperitoneally over 3 d to acclimate them to handling.

### CPP

**Figure 1.**
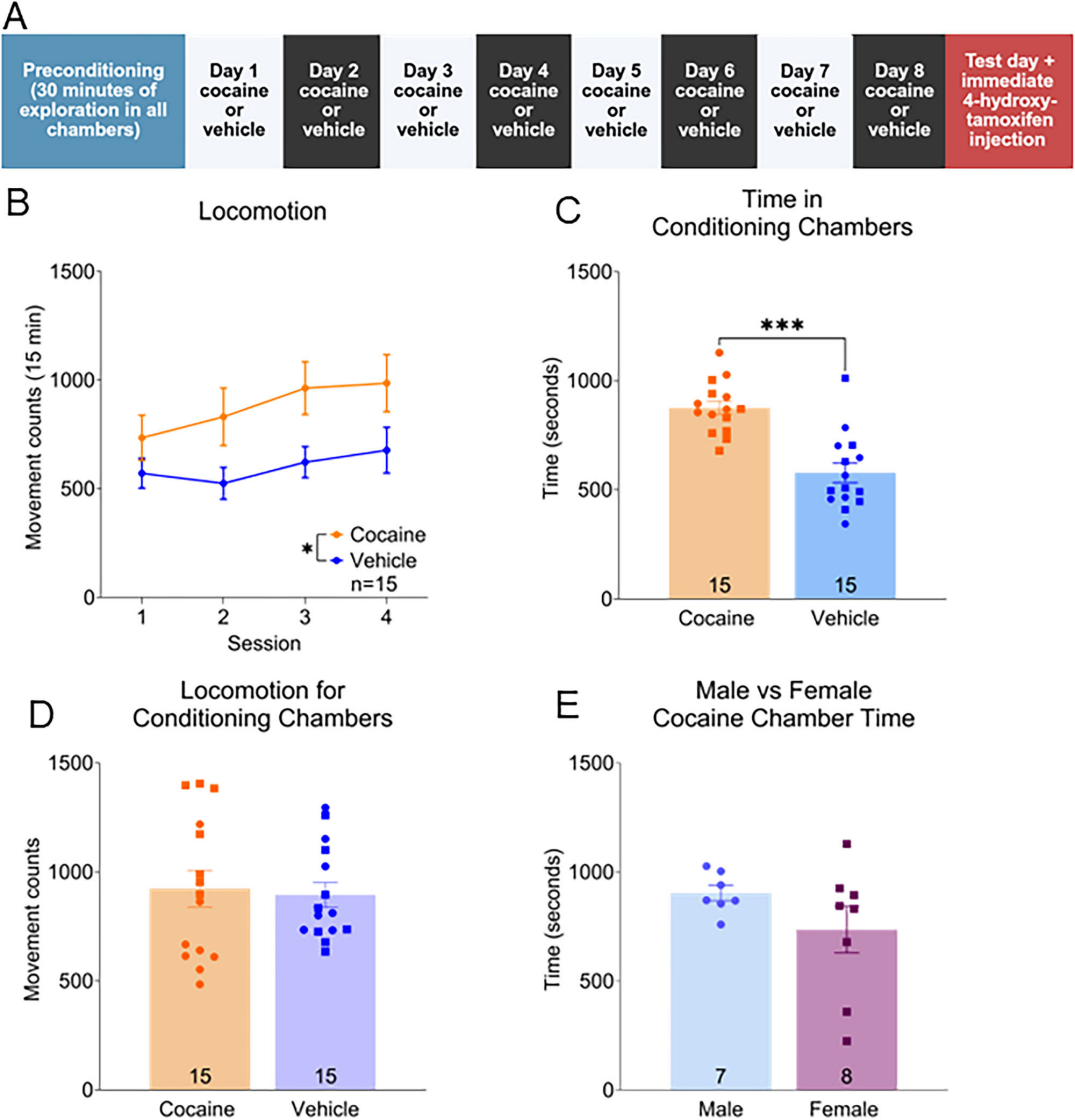
Cocaine CPP behavior. ***A***, Cocaine CPP timeline. Different colors denote different contexts. ***B***, Movement counts during conditioning sessions for the cocaine-paired chamber and the vehicle-saline chamber, two-way ANOVA. *p < 0.05. ***C***, Raw time in each chamber on the test day. ***p < 0.001. Comparing time in the cocaine-paired chamber to the vehicle-saline chamber, paired *t* test. ***D***, Locomotion of both chambers during test day, paired *t* test. ***E***, No sex differences in cocaine CPP. Numbers at the bottom of the bars indicate the sample size. Square data points represent females; circle data points represent males. See Extended Data Figures 1-1–1-4 for supporting analyses.

A three-chamber setup (Med Associates) with a white chamber with mesh flooring, a black chamber with rod flooring, and a gray connecting chamber was used for CPP experiments. On preconditioning days, mice were placed in the gray center chamber and allowed free access to all three chambers for 30 min as a pretest. Single-reward CPP experiments used a biased design in which each animal's least-preferred chamber (determined during pretest) was assigned as the reward-paired chamber. This approach has been described before (Cunningham et al., 2006; McKendrick and Graziane, 2020) and allows for testing whether cocaine or chocolate conditioning can shift preference and induce a robust place preference. In contrast, the dual cocaine–chocolate CPP experiments required a counterbalanced design. Because two rewards were compared within the same animals, half of the subjects received cocaine in their initially preferred chamber and half received chocolate in their preferred chamber. This ensured that neither reward was systematically paired with the preferred side by chance and that initial bias across groups was controlled for. Thus, the single-reward and dual-reward paradigms used different assignment strategies because they address different experimental questions. For single-reward experiments, on conditioning days, mice were confined to the less preferred chamber for 15 min during chocolate or cocaine administration and to the preferred chamber with vehicle (saline) on alternating days. Locomotor activity during conditioning sessions was quantified using the movement output from the Med Associates SOF-700RA-4 CPP program. This metric counts changes in the infrared beam broken within the active chamber zone, indexing within-zone positional shifts (e.g., traversing the chamber) while excluding micromovements and chamber-entry events (exploration/entrance), which are recorded separately. This continued for four conditioning sessions with the reward and four with the vehicle, as shown in Figure 1*A*. Mice were given vehicle injections before being placed into the chamber for both single- and dual-reward groups in cocaine experiments on noncocaine days. They were injected with 10 mg/kg of cocaine (NIDA Drug Supply Program) on cocaine-conditioning days. On chocolate-conditioning days, 10 miniature chocolate chips (Nestlé) were placed into a trough (one-half of an empty 50 ml Falcon conical tube; Corning) secured to the chamber floor; on vehicle days, an empty trough was used in the nonchocolate chamber. An additional “saline CPP” group was included to control for the experimental environment's impact on c-Fos expression for TRAPed cells, with mice in both chambers receiving saline injections across conditioning days. On test days, mice were placed into the gray center chamber and allowed to move freely through all three chambers for 30 min. Locomotor activity was recorded using the same movement metric described above. Immediately after the test session, mice received IP injections of 50 mg/kg 4-hydroxy-tamoxifen (4-OHT; RRID: SCR_013956, Sigma-Aldrich) prepared as previously described (DeNardo et al., 2019). Briefly, 10 mg of 4-OHT powder was dissolved in 250 μl of dimethyl sulfoxide (RRID: SCR_008426, Bio-Rad Laboratories) and frozen. Aliquots were thawed, mixed with 400 μl of 25% Tween 80 (RRID: SCR_013956, Sigma-Aldrich) and 4.35 ml of saline, and vortexed until clear, 5 min before the end of the test session. The 4-OHT was injected immediately after testing because the TRAP system labels neurons that are active within 6 h of administration (DeNardo et al., 2019), thereby minimizing off-target labeling. This timing is critical for activity-dependent tagging specificity, as activity-dependent gene expression occurs within a narrow window following neuronal activation, ensuring that neurons active during the test session are captured. Additionally, a control group received handling and saline intraperitoneal injections for 3 d before receiving a 50 mg/kg injection of 4-OHT in the home cage to control for basal c-Fos expression. An additional group went through cocaine CPP and was killed without receiving an injection of 4-OHT to confirm the necessity of 4-OHT administration to induce tdTomato^+^ expression. Because 4-OHT was administered immediately after the 30 min test, the TRAP approach captured neurons active throughout the entire test session.

### Immunohistochemistry

Mice were anesthetized with a 2:1 combination of ketamine (RRID: SCR_016162, MWI) and xylazine (RRID: SCR_016163, Akorn) and then perfused with phosphate-buffered saline (PBS; RRID: SCR_008492, Quality Biological) and 3.7% formaldehyde (RRID: SCR_008450, Thermo Fisher Scientific). Brains were postfixed in 3.7% formaldehyde for ≥24 h and then transferred to a solution of 20% sucrose (RRID: SCR_008454, Thermo Fisher Scientific) with 0.01% sodium azide (RRID: SCR_008453, Sigma-Aldrich) in 1× PBS. The 50 μm brain sections were cut using a cryostat (RRID: SCR_018884, Leica) from approximately bregma 2.0 to bregma 0.62. Free-floating sections were rinsed three times in 1× PBS and incubated for 2 h in a blocking solution of 5% normal goat serum (RRID: AB_2336990, Invitrogen), 2.5% bovine serum albumin (RRID: SCR_008445, Thermo Fisher Scientific), and 0.25% Triton X-100 (RRID: SCR_008453, Thermo Fisher Scientific) in PBS, then incubated in this blocking solution plus the primary antibody for 16–24 h. Anti-neuronal nuclei (NeuN) primary antibody (mouse, 1:1,000, RRID: AB_2298772, EMD Millipore Core) was used for neuron identification. The tissue was then rinsed one time in 0.25% PBS-Triton and three times in 1× PBS and incubated for 2 h in secondary antibodies. We used a 1:1,000 goat anti-mouse IgG (H + L) cross-absorbed secondary antibody conjugated to Alexa Fluor 647 (RRID: AB_2535814, Invitrogen, Thermo Fisher Scientific). The tissue was mounted using ProLong Gold (RRID: SCR_015961, Life Technologies).

### Image acquisition and quantification

Images of the NAcore with the anterior commissure and PL were obtained using a 63×, 1.4 NA oil immersion objective on a Zeiss 980 confocal microscope (Zeiss) or a Zeiss 880 confocal microscope (Zeiss) at the University of Wyoming or Colorado State University, respectively. The laser lines used were green (555 nm) for tdTomato and far-red (639 nm) for NeuN. For each mouse, 4–6 areas of both the NAcore and PL were selected and imaged. Each image was a 3 × 3 tile (512 × 512 pixels per image, 0.1444 mm^2^), with a *Z*-stack acquired at 1 μm steps, comprising 16–25 images per stack. Images were then stitched in the Zeiss Blue software (RRID: SCR_013672, Zeiss) and converted to the Imaris software (RRID: SCR_007370, Oxford Instruments) for image analysis. Before analysis, images were cropped to a standard *z*-thickness of 15 μm to ensure uniform image thickness. The NeuN nuclei (∼7 μm in diameter) were semiautomatically counted using the “spots” function and the mean intensity of the channel. A blinded experimenter manually checked and adjusted all automated counts. The tdTomato^+^ cell bodies (∼10 μm in diameter) were manually counted as TRAP2-labeled neurons. TdTomato^+^ cell density was calculated by dividing the number of tdTomato^+^ cells by the millimeter area per image, then averaging across images per animal. Normalized tdTomato expression was calculated by dividing the number of tdTomato^+^ cells by the number of NeuN cells per image, then averaging across images per animal. These normalized values are reported to complement raw tdTomato^+^ density measures and address group differences in NeuN labeling. One animal was removed from the single-cocaine experiment due to nondetectable tdTomato^+^ expression following conditioning. All ensemble measurements represent between-subject comparisons across experimental groups, as each animal contributed a single averaged tdTomato^+^ density measurement per region.

### Statistical analysis

All statistical analyses were performed using Prism 10 (GraphPad Software, RRID: SCR_002798). Numerical data were analyzed using appropriate parametric tests, with normality and homogeneity of variance assessed prior to analysis using the Shapiro–Wilk test. This study employed a nonrandom design to test whether the reward-paired context was preferred over the unpaired context by comparing raw time on the test day.

Behavioral results were analyzed using repeated-measures two–way ANOVA for within-subject factors (days and chamber) or paired/unpaired *t* tests, as appropriate. tdTomato^+^ cell density comparisons were conducted using a one-way ANOVA per brain region. Post hoc analyses were performed using Tukey's multiple-comparison tests to avoid pairwise bias. Regression analyses were used to assess correlations between tdTomato^+^ cell density and chamber preference. We also included a two-way ANOVA for each brain region, with sex as a factor.

Results are reported as mean ± SEM, with 95% confidence intervals (CIs) reported in Table 1. Outliers were identified using the ROUT method (Q = 0.5%) and removed before analysis. One mouse was removed from the single-reward cocaine ensemble group following outlier testing. Analysis of time spent in the gray chamber on test day was used to identify atypical behavioral patterns in mice, with a cutoff of 40% of the time spent in the gray chamber (Hnasko et al., 2007). One mouse in the saline group was excluded because it exceeded the 40% threshold in the gray chamber. Significance was defined as *p* < 0.05. Following ROUT analysis of tdTomato^+^ density in the NAcore, four images were removed from the cocaine group, four images from the chocolate group, three images from the dual cocaine–chocolate group, three images from the saline group, and two images from the home cage group. In the PL, one image was removed from the cocaine group, one image from the chocolate group, one image from the dual cocaine–chocolate group, and two images from the home cage group.

**Table 1.** Statistical summary table

Data structure	Type of test	CI
Normal distribution	RM two-way ANOVA (Fig. 1*B*)	95% CI [17.01, 620.5]
Normal distribution	Paired *t* test (Fig. 1*C*)	95% CI [154.7, 441.7]
Normal distribution	Paired *t* test (Fig. 1*D*)	95% CI [−268.2, 209.8]
Normal distribution	Unpaired *t* test (Fig. 1*E*)	95% CI [−426.0, 90.85]
Normal distribution	RM two-way ANOVA (Fig. 2*B*)	95% CI [−221.9, 157.2]
Normal distribution	Paired *t* test (Fig. 2*C*)	95% CI [−424.3, −67.34]
Normal distribution	Paired *t* test (Fig. 2*D*)	95% CI [−472.4, 29.27]
Normal distribution	Unpaired *t* test (Fig. 2*E*)	95% CI [−401.3, −51.84]
Normal distribution	RM two-way ANOVA (Fig. 3*B*)	95% CI [544.4, 1,610]
Normal distribution	Paired *t* test (Fig. 3*C*)	95% CI [−22.91, −3.20]
Normal distribution	Paired *t* test (Fig. 3*D*)	95% CI [−707.5, 13.32]
Normal distribution	Unpaired *t* test (Fig. 3*E*)	95% CI [−6.75, 13.76]
Normal distribution	One-way ANOVA (Fig. 4*C*)	Cocaine 4-OHT^−^ versus saline
		95% CI [−49.90, −1.042]
		Cocaine 4-OHT^−^ versus 4-OHT^+^
		95% CI [−54.84, −5.989]
Normal distribution	One-way ANOVA (Fig. 4*D*)	Home cage
		Versus cocaine 4-OHT^+^ 95% CI [−129.2, −33.47]
		Versus chocolate 95% CI [−136.0, −42.03]
		Versus dual 95% CI [−124.6, −26.06]
		Cocaine 4-OHT^−^
		Versus saline 95% CI [−80.19, −7.085]
		Versus cocaine 4-OHT^+^ 95% CI [−117.9, −44.77]
		Versus chocolate 95% CI [−124.5, −53.60]
		Versus dual 95% CI [−113.8, −36.95]
		Saline
		Versus cocaine 4-OHT^+^ 95% CI [−65.32, −10.06]
		Versus chocolate 95% CI [−71.54, −19.27]
		Versus dual 95% CI [−61.75, −1.675]
Normal distribution	Two-way ANOVA (Extended Data Fig. 1-1*B*)	95% CI [257.8, 416.6]
Normal distribution	Paired *t* test (Extended Data Fig. 1-1*C*)	95% CI [−80.73, 306.0]
Normal distribution	Paired *t* test (Extended Data Fig. 1-1*D*)	95% CI [−432.9, −147.8]
Normal distribution	Unpaired *t* test (Extended Data Fig. 1-1*E*)	95% CI [−346.0, 6.43]
Normal distribution	Two-way ANOVA (Fig. 5*A*)	95% CI [−8.32, 12.94]
Normal distribution	Two-way ANOVA (Fig. 5*B*)	95% CI [−14.23, 17.60]
Normal distribution	RM two-way ANOVA (Extended Data Fig. 1-4*B*)	95% CI [−462.5, 1,540]
Normal distribution	Paired *t* test (Extended Data Fig. 1-2*C*)	95% CI [−584.8, −219.8]
Normal distribution	Paired *t* test (Extended Data Fig. 1-2*D*)	95% CI [−1,195, 240.7]
Normal distribution	Unpaired *t* test (Extended Data Fig. 1-2*E*)	95% CI [−18.92, 6.018]
Normal distribution	Simple linear regressions (Extended Data Fig. 1-3)	NAcore 95% CI [−0.30, 0.67]
		PL 95% CI [−0.61, 0.39]
Normal distribution	Simple linear regressions (Extended Data Fig. 1-4)	NAcore 95% CI [−0.72, 0.22]
		PL 95% CI [−0.39, 0.62]
Normal distribution	Simple linear regressions (Extended Data Fig. 3-1)	NAcore 95% CI [−0.85, 0.12]
		PL 95% CI [−0.61, 0.59]
Normal distribution	Simple linear regressions (Extended Data Fig. 2-1)	NAcore 95% CI [0.11, 0.78]
		PL 95% CI [−0.11, 0.70]
Normal distribution	One-way ANOVA (Extended Data Fig. 4-1*A*)	Cocaine 4-OHT−
		Versus home cage 95% CI [789.3, 3,909]
		Versus cocaine 4-OHT^+^ 95% CI [−2,372, −241.5]
		Versus chocolate 95% CI [−2,535, −469.0]
		Versus dual 95% CI [−3,603, −1,364]
		Dual
		Versus saline 95% CI [−2,377, −625.3]
		Versus cocaine 4-OHT^+^ 95% CI [−2,052, −301]
		Versus chocolate 95% CI [−1,817, −145.9]
Normal distribution	One-way ANOVA (Extended Data Fig. 4-1*B*)	Cocaine 4-OHT^−^
		Versus cocaine 4-OHT^+^ 95% CI [−1,994, 137.5]
		Versus chocolate 95% CI [−2,598, −798.4]
		Versus dual 95% CI [−2,301, −350.3]
		Saline
		Versus chocolate 95% CI [−1,527, −199.1]
Normal distribution	One-way ANOVA (Extended Data Fig. 4-2*A*)	Home cage
		Versus saline 95% CI [0.05, 0.96]
		Versus cocaine 4-OHT^+^ 95% CI [0.12, 1.03]
		Versus chocolate 95% CI [0.14, 1.03]
		Versus dual 95% CI [0.02, 0.96]
		Cocaine 4-OHT^−^
		Versus saline 95% CI [0.16, 0.85]
		Versus cocaine 4-OHT^+^ 95% CI [0.23, 0.92]
		Versus chocolate 95% CI [0.25, 0.93]
		Versus dual 95% CI [0.12, 0.85]
Normal distribution	One-way ANOVA (Extended Data Fig. 4-2*B*)	Home cage
		Versus saline 95% CI [0.56, 2.97]
		Versus cocaine 4-OHT^+^ 95% CI [1.53, 3.95]
		Versus chocolate 95% CI [1.24, 3.62]
		Versus dual 95% CI [1.05, 3.54]
		Cocaine 4-OHT^−^
		Versus saline 95% CI [0.85, 2.68]
		Versus cocaine 4-OHT^+^ 95% CI [1.82, 3.66]
		Versus chocolate 95% CI [1.24, 3.62]
		Versus dual 95% CI [1.33, 3.27]
		Saline
		Versus cocaine 4-OHT^+^ 95% CI [0.29, 1.67]
		Versus chocolate 95% CI [0.02, 1.31]

The table reports the structure, statistical test used, and 95% CIs for all analyses. Test statistics (*F*, *t*, degrees of freedom) and *p* values are reported in the Results section. Figure and extended data figure references indicate where each analysis appears. Repeated-measures (RM).

## Results

### Single-cocaine CPP behavior

We conducted a behavioral assay for cocaine-seeking behavior in a standard single-reward CPP (Fig. 1*A*). During conditioning, c-Fos-TRAP2 mice showed increased locomotion in the cocaine-paired chamber (Fig. 1*B*; *F*_(1, 14)_ = 5.13; *p* = 0.0399). On test day, mice showed a significant preference for the cocaine-paired chamber compared with the chamber conditioned with saline vehicle (Fig. 1*C*; *t*_(14)_ = 4.457; *p* = 0.0005; cocaine chamber 875.7 ± 30.69 s; vehicle chamber 577.5 ± 44.56 s). Test-day locomotion did not differ between chambers (Fig. 1*D*; *t*_(14)_ = 0.26; *p* = 0.80). No sex differences were observed (Fig. 1*E*; *n*_male_ = 7; *n*_female_ = 8; *t*_(13)_ = 1.40; *p* = 0.1847; male 903.9 ± 35.17 s; female 736.4 ± 107.1 s).

10.1523/ENEURO.0463-25.2026.f1-1 Figure 1-1 Saline CPP behavior. (**A**) Saline CPP timeline. Different colors denote different contexts. (**B**) Movement counts during both black and white conditioning sessions. **** p < 0.0001 Comparing black and white chamber movement conditioning. (**C**) Raw time during test day for the different contexts, paired t-test. (**D**) Locomotion of both chambers during test day, *** p < 0.001, paired t-test. (**E**) Sex differences in context preference were not observed, unpaired t-test. Numbers at the bottom of the bars indicate the sample size. Square data points represent females; circle data points represent males. Download Figure 1-1, TIF file.

**Figure 2.**
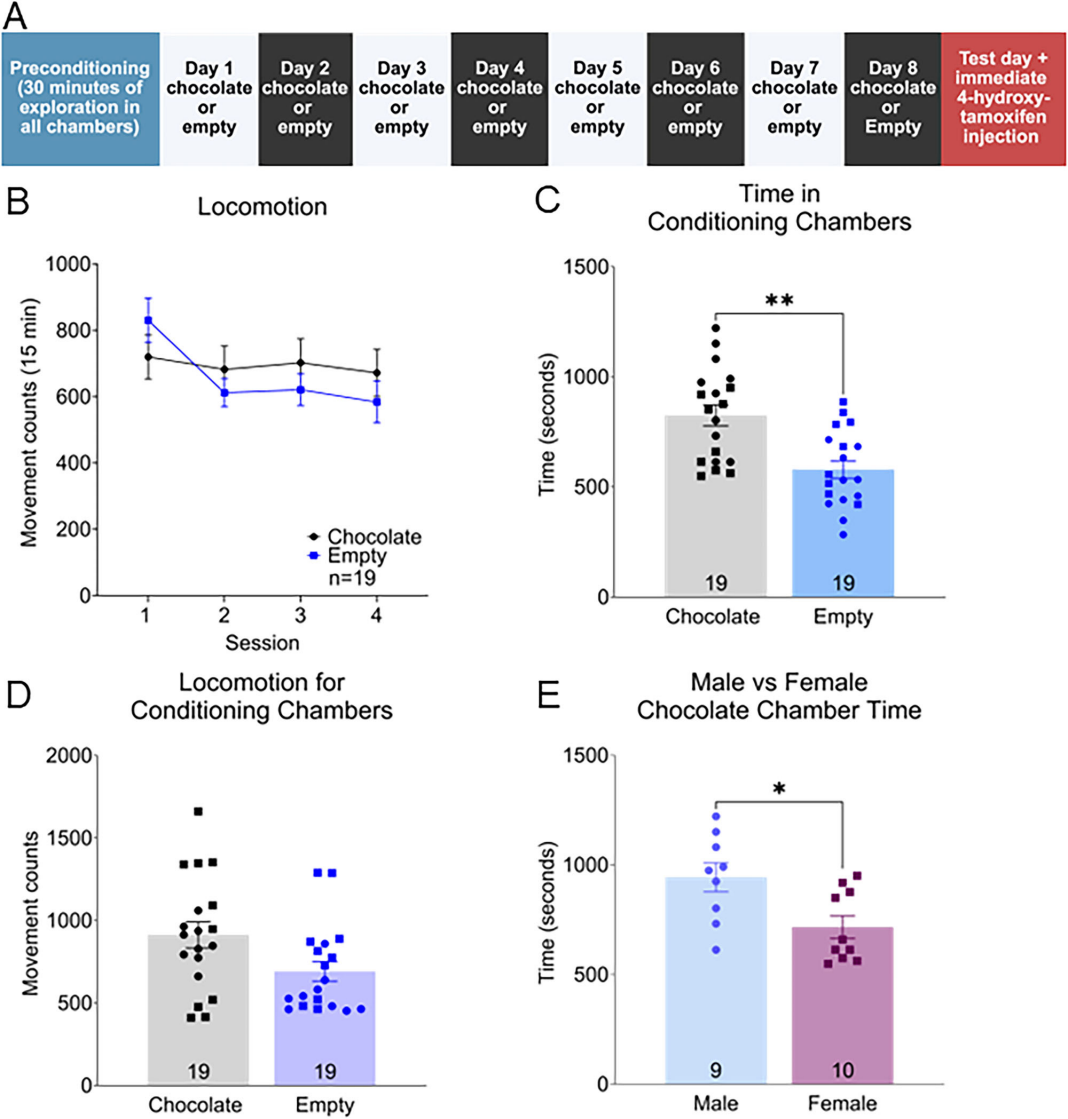
Chocolate CPP behavior. ***A***, Chocolate CPP timeline. Different colors denote different contexts. ***B***, Movement counts during conditioning sessions for the chocolate-paired and vehicle-empty chambers, two-way ANOVA. ***C***, Raw time in each chamber on the test day. **p < 0.01 Comparing time in the chocolate-paired chamber to the empty-paired chamber, paired *t* test. ***D***, Locomotion of both chambers during test day, paired *t* test. ***E***, Sex differences in chocolate-chamber time revealed that male mice spent more time in the context than female mice. *p < 0.05. Comparing chocolate chamber time between sexes, unpaired *t* test. Numbers at the bottom of the bars indicate the sample size. Square data points represent females; circle data points represent males. See Extended Data Figure 2-1 for supporting analysis.

10.1523/ENEURO.0463-25.2026.f1-2 Figure 1-2 Cocaine CPP without 4-hydroxy-tamoxifen (4-OHT) behavior. (**A**) Cocaine CPP 4-OHT- timeline. Different colors denote different contexts. (**B**) Movement counts during conditioning sessions for the cocaine-paired chamber and the vehicle-saline chamber. (**C**) Raw time in each chamber on test day. ** p < 0.01 Comparing time in the cocaine-paired chamber to the vehicle-saline chamber, paired t-test. (**D**) Locomotion of both chambers during test day, paired t-test. (**E**) Males and females differ in time spent in the cocaine-paired chamber * p < 0.05, unpaired t-test. Numbers at the bottom of the bars indicate the sample size. Square data points represent females; circle data points represent males. Download Figure 1-2, TIF file.

**Figure 3.**
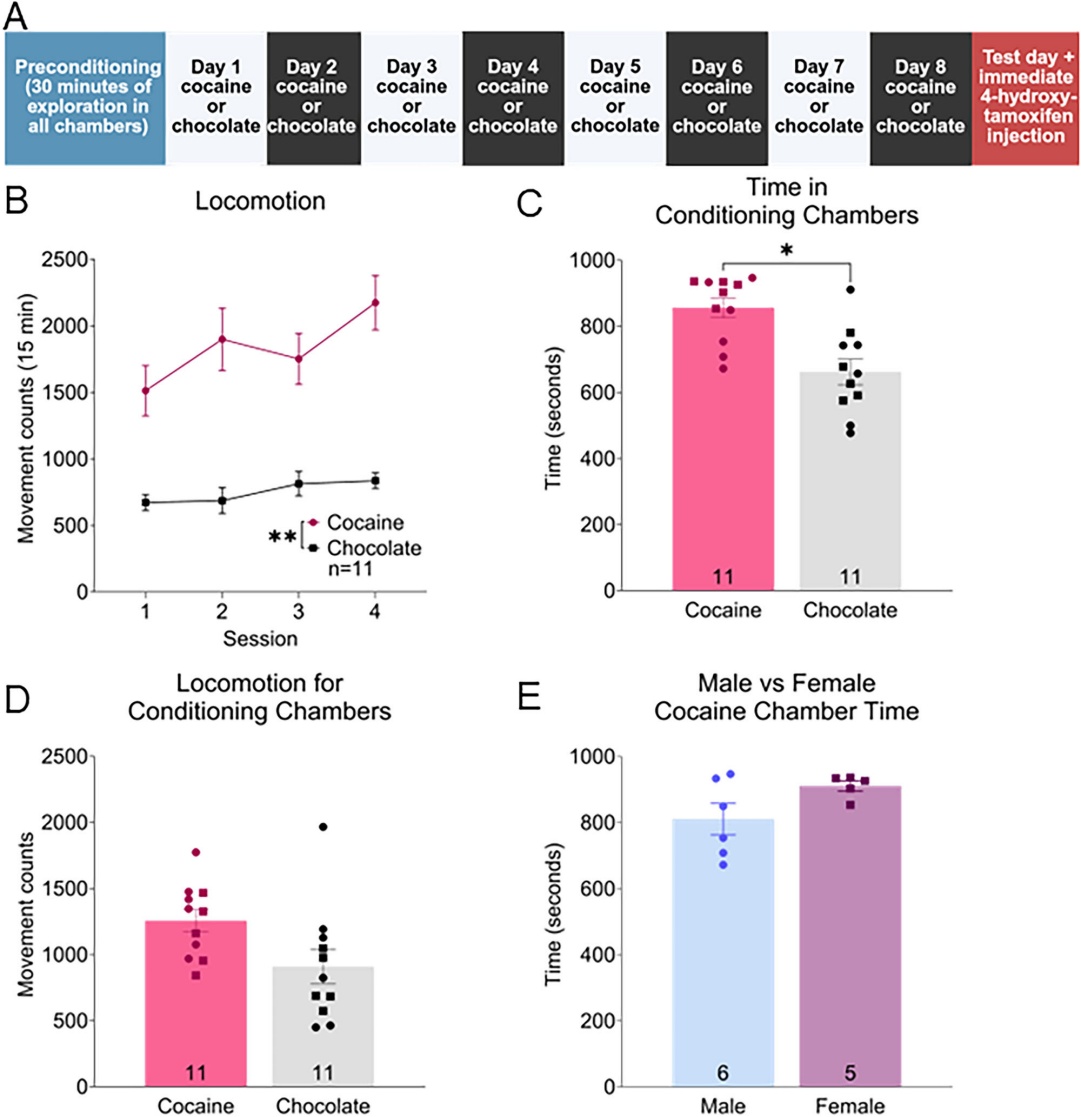
Dual cocaine–chocolate CPP. ***A***, Dual cocaine–chocolate CPP timeline. Different colors denote different contexts. ***B***, Movement counts during conditioning sessions for the cocaine-paired chamber and the chocolate-paired chamber. **p < 0.01. Comparing movement counts during conditioning, two-way ANOVA. ***C***, Raw time in each chamber on the test day. *p < 0.05. Comparing the cocaine-paired chamber and the chocolate-paired chamber, paired *t* test. ***D***, Locomotion of both chambers during test day, paired *t* test. ***E***, No sex differences in dual CPP for cocaine-paired contexts were found, unpaired *t* test. Numbers at the bottom of the bars indicate the sample size. Square data points represent females; circle data points represent males. See Extended Data Figure 3-1 for supporting analysis.

10.1523/ENEURO.0463-25.2026.f1-3 Figure 1-3 Correlations between **c**ocaine-chamber time and tdTomato + cell density. (**A**) Correlation of cocaine-chamber time with the nucleus accumbens core (NAcore) tdTomato + cell density. (**B**) Correlation of cocaine-chamber time with the prelimbic cortex (PL) tdTomato + cell density**.** Download Figure 1-3, TIF file.

**Figure 4.**
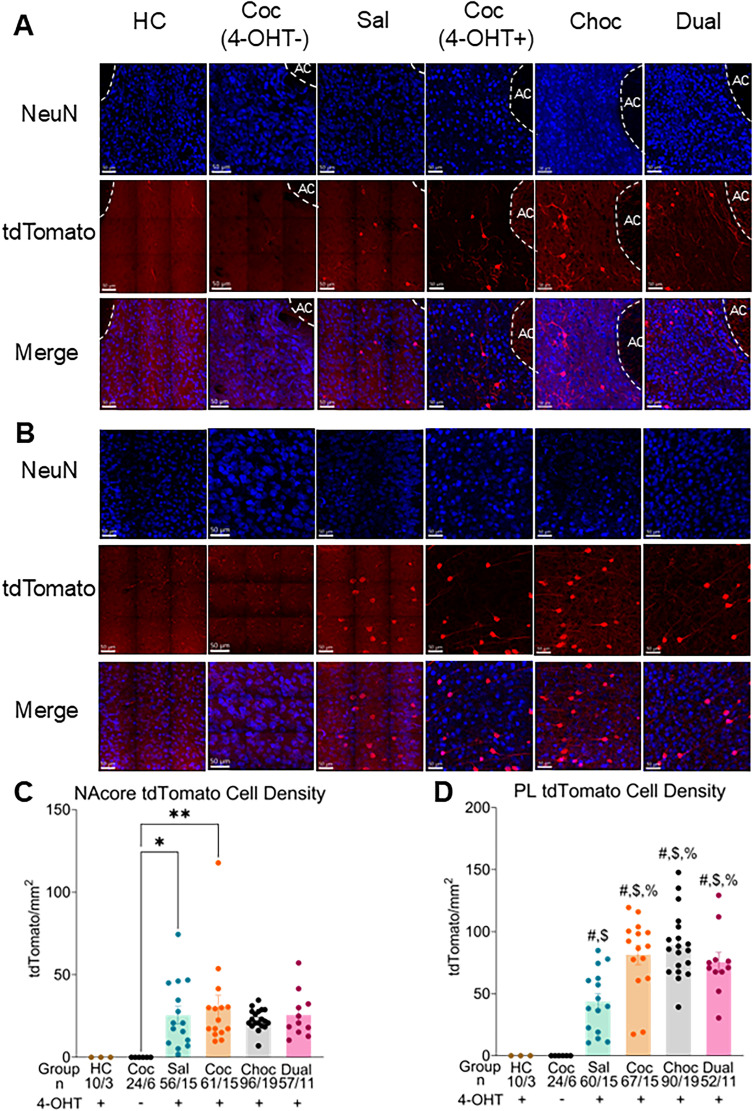
tdTomato^+^ area following single- and dual-reward CPP. ***A***, Representative images of the nucleus accumbens core (NAcore). Anterior commissure (AC), home cage (HC), cocaine (Coc), 4-hydroxy-tamoxifen (4-OHT), saline (Sal), chocolate (Choc), dual cocaine and chocolate (Dual). ***B***, Representative images of the prelimbic cortex (PL). ***C***, Area counts of the context ensemble NAcore within experimental groups. *p < 0.05 compared with cocaine 4-OHT^−^. **p < 0.01 compared to cocaine 4-OHT^−^ ***D***, Size of the context ensemble in the PL across reward groups. ^#^*p* < 0.05 compared to PL HC; ^$^*p* < 0.05 compared with PL Coc (4-OHT^−^), ^%^*p* < 0.05 compared with PL Sal. Numbers indicate images over behavioral sample size. See Extended Data Figures 4-1 and 4-2 for supporting analysis.

10.1523/ENEURO.0463-25.2026.f1-4 Figure 1-4 Correlations between saline locomotion and tdTomato + cell density. **(A**) Correlation of locomotion with the nucleus accumbens core (NAcore) tdTomato + cell density. (**B**) Correlation of locomotion with the prelimbic cortex (PL) tdTomato + cell density. Download Figure 1-4, TIF file.

### Single chocolate CPP behavior

A total of 19 c-Fos-TRAP2 mice trained in the single chocolate CPP (Fig. 2*A*) showed no difference in locomotion during conditioning (Fig. 2*B*; *F*_(1,18)_ = 0.1287; *p* = 0.7240). Following conditioning, mice significantly preferred the chocolate-paired chamber on test day compared with the chamber in which chocolate was never presented (Fig. 2*C*; *t*_(18)_ = 2.894; *p* = 0.0097; chocolate chamber 824.4 ± 48.23 s; empty chamber 578.6 ± 39.67 s). Test-day locomotion did not differ between chambers (Fig. 2*D*; *t*_(18)_ = 1.86; *p* = 0.0799). Males displayed a stronger preference for the chocolate-paired chamber than females (Fig. 2*E*; *n*_male_ = 9; *n*_female_ = 10; *t*_(17)_ = 2.736; *p*=0.0141; male 943.6 ± 66.35 s; female 717.1 ± 51.14 s).

10.1523/ENEURO.0463-25.2026.f2-1 Figure 2-1 Correlations between chocolate-chamber time and tdTomato + cell density (**A**) Correlation of chocolate-chamber time with the nucleus accumbens core (NAcore) tdTomato + cell density. (**B**) Correlation of chocolate-chamber time with the prelimbic (PL) tdTomato + cell density. Download Figure 2-1, TIF file.

### Dual cocaine–chocolate CPP behavior

In our paradigm of dual cocaine–chocolate CPP, we alternated cocaine and chocolate days in distinctive chambers during conditioning (Fig. 3*A*). During conditioning, the 11 c-Fos-TRAP2 mice included in the experiment exhibited greater locomotion in the cocaine-paired chamber than in the chocolate-paired chamber (Fig. 3*B*; *F*_(1, 10)_ = 20.87; *p* = 0.0010). On test day, mice significantly preferred the cocaine-paired chamber to the chocolate-paired chamber (Fig. 3*C*; *t*_(10)_ = 2.962; *p* = 0.0142; cocaine chamber 856.3 ± 30.16 s; chocolate chamber 662.2 ± 38.56 s). Test-day locomotion did not differ between chambers (Fig. 3*D*; *t*_(10)_ = 2.146; *p* = 0.0575). No sex differences were observed between groups (Fig. 3*E*; *n*_male_ = 6; *n*_female_ = 5; *t*_(9)_ = 1.898; *p* = 0.0993; male cocaine 810.8 ± 47.61 s; female 910.8 ± 15.41 s). Because mice freely explored both reward-paired chambers during the 30 min test, the TRAP2-labeled population in the dual-CPP group reflects a mixture of neurons from both cocaine- and chocolate-associated contexts, preventing inference about the relative contribution of cocaine- versus chocolate-associated activity. Thus, while the ensemble is biased toward cocaine-context activity, it cannot be interpreted as exclusively cocaine specific.

10.1523/ENEURO.0463-25.2026.f3-1 Figure 3-1 Correlation between cocaine-chamber time in dual conditioning with tdTomato + cell density. (**A**) Correlation of cocaine-chamber time in dual conditioning with nucleus accumbens core (NAcore) tdTomato + cell density. (**B**) Correlation of **cocaine-chamber time** in dual conditioning with the prelimbic cortex (PL) tdTomato + cell density. Download Figure 3-1, TIF file.

### Characterization of TRAP2-labeled neurons across experimental groups

To compare TRAP2-labeled tdTomato^+^ neuron counts across regions and experimental groups, we quantified tdTomato^+^ fluorescence signals. Before this, to validate that TRAP2 labeling reflected neuronal activity during the test session, a control group of 15 c-Fos-TRAP2 mice underwent saline CPP in both chambers without exposure to any other reward and received a 4-OHT injection immediately following the test session (Extended Data Fig. 1-1*A*). Locomotion measurements during conditioning revealed a significant difference between the black and white chambers, with greater movement observed in the black chamber than in the white (Extended Data Fig. 1-1*B*; *F*_(1, 14)_ = 82.95; *p* < 0.0001). A probable explanation is differences in flooring: mice prefer solid-bar flooring to mesh (CPP Contextual Floor, n.d.). As expected, no chamber preference was observed on test day (Extended Data Fig. 1-1*C*; *t*_(14)_ = 1.249; *p* = 0.2320; white chamber 735.6 ± 50.41 s; black chamber 623.0 ± 45.28 s). However, there was a difference in locomotion (Extended Data Fig. 1-1*D*; *t*_(14)_ = 4.368; *p* = 0.0006). Sex differences were also absent between males and females, showing similar black-chamber preference (Extended Data Fig. 1-1*E*; *n*_male_ = 8; *n*_female_ = 7; *t*_(13)_ = 2.082; *p* = 0.0577; male 702.2 ± 67.69 s; female 532.4 ± 39.73 s).

To validate that TRAP2 tagging is 4-OHT-dependent, six animals went through cocaine CPP with no 4-OHT injection on test day, and animals were instead sacrificed on test day (Extended Data Fig. 1-2*A*). During conditioning, mice did not exhibit the typical increase in locomotion during cocaine sessions (Extended Data Fig. 1-2*B*; *F*_(1,5)_ = 3.300; *p* = 0.1276). However, on test day, mice significantly preferred the cocaine-paired chamber (Extended Data Fig. 1-2*C*; *t*_(5)_ = 5.667; *p* = 0.0024; cocaine chamber, 902.6 ± 41.77 s; vehicle chamber, 500.2 ± 36.99 s), with no difference in locomotion (Extended Data Fig. 1-2*D*; *t*_(5)_ = 1.709; *p* = 0.1482). Comparing males and females revealed that males spend more time in the cocaine chamber (Extended Data Fig. 1-2*E*; *n*_male_ = 3; *n*_female_ = 3; *t*_(4)_ = 4.420; *p* = 0.0115; male cocaine 987.7 ± 20.30 s; female cocaine 817.4 ± 32.72 s). Given the small sample size (*n* = 3 per sex), these analyses are underpowered and should be interpreted descriptively rather than inferentially. Following tissue collection, confocal imaging confirmed no tdTomato^+^ expression in neuronal somas in the sampling of the NAcore (Fig. 4*A*) or PL (Fig. 4*B*).

10.1523/ENEURO.0463-25.2026.f4-1 Figure 4-1 NeuN density across groups. (**A**) Expression of NeuN in the nucleus accumbens core (NAcore) differed across groups: home cage (HC), cocaine (Coc), saline (Sal), chocolate (Choc), cocaine and chocolate (dual), and 4-hydroxy-tamoxifen (4-OHT). $ p < 0.01 compared to cocaine 4-OHT-, & p < 0.05 compared to dual. **(B)** NeuN expression in the prelimbic cortex (PL) differed across groups. # p < 0.01 compared to saline, $ p < 0.05 compared to cocaine 4-OHT-. Numbers indicate images over behavioral sample size. Download Figure 4-1, TIF file.

10.1523/ENEURO.0463-25.2026.f4-2 Figure 4-2 tdTomato expression normalized to NeuN. (**A**) Percent tdTomato+/ NeuN + cells in the nucleus accumbens core, home cage (HC), cocaine (Coc), chocolate (Choc), cocaine and chocolate (dual), and 4-hydroxytamoxifen (4-OHT). # p < 0.0001 compared to HC. (**B**) Percent tdTomato+/ NeuN + cells in the PL across reward groups. $ p < 0.0001 compared to saline. Numbers indicate the number of image acquisitions followed by behavioral sample size. Download Figure 4-2, TIF file.

We next examined group differences in tdTomato^+^ cell density in two key regions: the NAcore and the PL. Representative images of NAcore and PL are shown in Figure 4, *A* and *B*, respectively. When comparing tdTomato-labeled cell density in the NAcore, we observed differences across experimental groups (Fig. 4*C*; *F*_(5,63)_ = 3.876; *p* = 0.0040). Tukey's post hoc analysis revealed a difference between the cocaine CPP 4-OHT^−^ group and the saline (*p* = 0.0361) and cocaine CPP 4-OHT^+^ (*p* = 0.0066). Comparing tdTomato^+^ cell density in the PL across groups revealed significant differences (Fig. 4*D*; *F*_(5, 63)_ = 18.38; *p* < 0.0001). Post hoc testing revealed that the home cage group differed from the cocaine 4-OHT^+^ CPP (*p* < 0.0001), chocolate CPP (*p* < 0.0001), and cocaine in the dual-CPP group (*p* = 0.0004), but not from the saline or cocaine CPP 4-OHT^−^. When compared with the cocaine CPP 4-OHT^−^ group, there was a difference with saline CPP (*p* = 0.0104), cocaine CPP 4-OHT^+^, chocolate CPP, and cocaine in the dual-CPP group (*p* < 0.0001). Saline CPP animals were different from cocaine CPP 4-OHT^+^ (*p* = 0.0022), chocolate CPP (*p* < 0.0001), and cocaine in the dual-CPP group (*p* = 0.0326). There were no differences in tdTomato^+^ density among the cocaine, chocolate, and dual-CPP groups.

**Figure 5.**
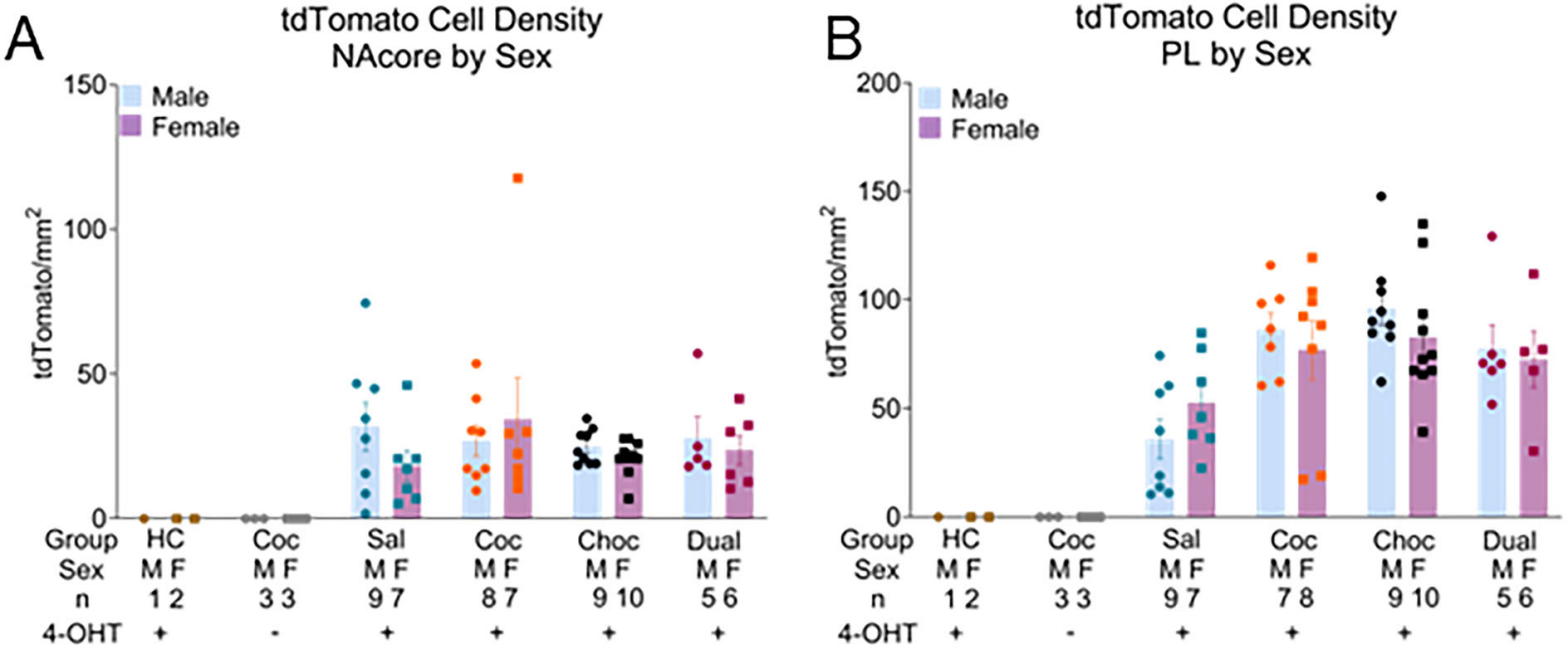
tdTomato^+^ area following single- and dual-reward CPP by sex. ***A***, Nucleus accumbens core (NAcore) tdTomato^+^ area by sex. Home cage (HC), saline (Sal), cocaine (Coc), chocolate (Choc), and dual cocaine and chocolate (Dual). 4-OHT. ***B***, Prelimbic cortex (PL) tdTomato^+^ area by sex. Sex was included as a factor in the statistical analysis; no significant main effect of sex or reward × sex interaction was detected in either region. Numbers indicate the behavioral sample size.

We next examined whether biological sex influenced the number of TRAP2-labeled neurons. Two-way ANOVA (reward group × sex) analysis revealed a significant main effect of reward group on tdTomato^+^ cell density in the NAcore (Fig. 5*A*; *F*_(5, 57)_ = 3.620; *p* = 0.0065), with no main effect of sex or a reward-by-sex interaction. A similar pattern was observed in the PL (Fig. 5*B*), where a significant main effect of the reward group was detected (*F*_(5, 57)_ = 17.18; *p* < 0.0001), with no effect of sex and no interaction between reward group and sex. These results indicated that differences in tdTomato^+^ cell density across groups were not significantly influenced by sex in either region.

To assess whether tdTomato^+^ cell density in the NAcore and PL was related to chamber preference, we performed simple linear regressions of seconds in the reward chamber on tdTomato^+^ cell density. We found no significant relationship between tdTomato^+^ cell density and time spent in the cocaine-paired chamber for either NAcore or PL (NAcore, Extended Data Fig. 1-3*A*; simple linear regression, *R*^2^ = 0.06166; *p* = 0.3722; PL, Extended Data Fig. 1-3*B*, *R*^2^ = 0.02226; *p* = 0.5957), nor cocaine seeking in dual-reward conditioning (NAcore, Extended Data Fig. 3-1*A*; *R*^2^ = 0.2659; *p* = 0.1044; PL, Extended Data Fig. 3-1*B*; *R*^2^ = 0.0004; *p* = 0.9502). In the chocolate CPP group, tdTomato^+^ cell density in the NAcore significantly correlated with time spent in the chocolate-paired chamber in the NAcore, but not the PL (NAcore, Extended Data Fig. 2-1*A*; *R*^2^ = 0.2893; *p* = 0.0175; PL, Extended Data Fig. 2-1*B*; *R*^2^ = 0.1292; *p* = 0.1307). To ensure that behavioral movements were not correlated to tdTomato^+^ cell density, we also assessed total locomotion to tdTomato^+^ density in the saline CPP animals. We found no correlation (NAcore, Extended Data Fig. 1-4*A*; simple linear regression, *R*^2^ = 0.1095; *p* = 0.2284; PL, Extended Data Fig. 1-4*B*; *R*^2^ = 0.02504; *p* = 0.5732). These analyses revealed no consistent relationship between tdTomato^+^ cell density and chamber preference or locomotion.

To assess potential variability in neuronal counts, we quantified NeuN cell density across experimental groups. We found significant differences in cell density between the NAcore (Extended Data Fig. 4-1*A*; *F*_(5,63)_ = 10.76; *p* < 0.0001) and PL (Extended Data Fig. 4-1*B*; *F*_(5,63)_ = 7.291; *p* < 0.0001). This indicated that NeuN staining varied across groups, meaning that tdTomato^+^ cell density was a more reliable metric than percent tdTomato^+^ over NeuN^+^ cells in the images for comparisons. To confirm that this variation in NeuN labeling did not bias the interpretation of tdTomato^+^ density metrics by altering the number of tdTomato^+^ cells observed, we normalized our data to the percentage of tdTomato^+^ cells among NeuN^+^ cells. We found significant differences in the NAc (Extended Data Fig. 4-2*A*; *F*_(5,63)_ = 8.172; *p* < 0.0001) and the PL (Extended Data Fig. 4-2*B*; *F*_(5,63)_ = 23.42; *p* < 0.0001). Importantly, these differences preserved the overall pattern of group comparisons observed in the tdTomato^+^ density analysis, indicating that the observed ensemble differences were not solely attributable to variability in NeuN labeling or neuronal density across groups.

## Discussion

We used a dual-reward paradigm for neuronal ensemble tagging in a noncontingent dual model. Mice exhibited a preference for the cocaine- and chocolate-paired chambers following single-reward conditioning. This finding aligns with previous work demonstrating that noncontingent models effectively assess preference for rewarding stimuli (Orsini et al., 2013; O’Neal et al., 2022). When conditioned with both cocaine and chocolate, mice consistently preferred the cocaine-paired chamber on test day, indicating that cocaine-associated contextual cues exerted stronger conditioned influence than chocolate-associated contextual cues in this CPP paradigm when no reward was present. We examined tdTomato^+^ cell density in neurons tagged during exposure to cocaine- and chocolate-paired contexts in the NAcore and PL following single- and dual-reward CPP training. TRAP2-labeled tdTomato^+^ cell density was consistent within each region across experimental reward conditions; however, only the chocolate CPP group in the NAcore showed a significant correlation with time spent in the reward chambers.

In this project, we use the term “ensemble” to refer to neurons tagged via TRAP2 during the 30 min test session, reflecting activity during exploration of both contexts rather than isolated, functionally defined reward-seeking ensembles. In the dual-reward paradigm, animals traverse both chambers during the 30 min test, and the TRAP2 window captures activity related to exploration, contextual processing, and exposure to both contexts. Because animals explored both chambers during the test session, with the TRAP2 window integrating activity across the full 30 min, the tagged population represents a mixed ensemble reflecting activity in both reward-paired contexts. This design does not permit inference about the proportional contribution of cocaine- versus chocolate-associated activity. Importantly, because the present study did not manipulate TRAP2-labeled populations, these findings cannot determine whether these neuronal populations are causally required for reward-related behaviors.

Although no sex differences were observed overall in the dual cocaine–chocolate CPP, males showed a greater preference for the chocolate chamber compared with females in the single-reward group (Fig. 2*E*). This finding contradicts previous reports of higher consumption of highly palatable food in female rats compared with male rats under low-cost conditions, with no differences in consumption observed at higher costs (Freeman et al., 2021). In this context, “low cost” refers to conditions in which access to food requires minimal effort, while “high cost” refers to conditions that require greater effort. This study by Freeman et al. utilized a within-session behavioral–economic paradigm, a contingent model in which active effort is required to receive food. In contrast, our approach employs a noncontingent model where food is provided independently of effort. These methodological differences may account for discrepancies in consumption across studies.

In the dual cocaine–chocolate CPP, mice preferred the cocaine-paired chamber (Fig. 3*C*). This behavioral preference suggests that cocaine-paired contextual cues exert a stronger conditioned influence relative to natural rewards when only the reward context is presented. Such a preference indicates that, under noncontingent conditions, cocaine-paired contextual cues dominate over chocolate-paired ones. These findings are consistent with previous literature on the seeking of cocaine in contingent SA models in the absence of rewards (Tunstall and Kearns, 2016; Bobadilla et al., 2020). This differs from paradigms in which rats overwhelmingly chose nondrug sweetened water over cocaine when the actual rewards are delivered after the choice (Lenoir et al., 2007). This preference was later attributed to differences in the pharmacokinetics of dopamine release between rewards (Wise and Koob, 2014; Canchy et al., 2021).

In previous studies comparing stimulants to socialization, several findings stand out. Using a CPP paradigm, only four 15 min episodes of dyadic social interaction (mutual social interaction between two subjects) with a sex- and weight-matched conspecific were sufficient to reverse CPP from cocaine to social interaction despite continued cocaine training and prevent the reinstatement of cocaine CPP (Zernig et al., 2013; Zernig and Pinheiro, 2015; Bregolin et al., 2017). Neuroanatomical manipulations further support this shift: inactivation of the NAcore or the basolateral amygdala redirected CPP away from cocaine toward social interaction, whereas lesioning the nucleus accumbens shell produced the opposite effect (Fritz et al., 2011; Zernig and Pinheiro, 2015). Examining the nucleus accumbens corridor (from the interhemispheric sulcus to the anterior commissure) for receptor types active in response to concurrent cocaine socialization CPP revealed a preference for D1 receptor-expressing neurons to be active following CPP testing, which was reduced both behaviorally and neurobiologically by socialization (Prast et al., 2014). These findings highlight that both receptor-level mechanisms and environmental factors shape the balance between drug and social rewards. For example, the preference for social interaction over stimulant reward is strongly influenced by housing conditions, with individually housed animals typically showing greater sensitivity to social reward than their group-housed counterparts (Yates et al., 2013). Importantly, within natural reward paradigms, social interaction differs fundamentally from ingestible rewards such as sugar or chocolate: the former is an experiential, relational stimulus, whereas the latter is a consumable, hedonic stimulus. While social interaction is rewarding, it is often considered experiential rather than purely hedonic because it engages complex relational and emotional processes beyond simple sensory pleasure (Matyjek et al., 2020). The findings of the present study, showing a preference for the chamber paired with the drug reward (cocaine) over the chamber paired with the natural reward (chocolate), may reflect differences in reward salience between social rewards and highly palatable food (Chow et al., 2022).

We observed no significant differences between male and female mice in the dual cocaine–chocolate CPP (Fig. 3*D*); both sexes sought out the cocaine-associated context more than the chocolate context. Importantly, this was true even when the animals were food-deprived during conditioning and testing. It is known that placing animals in a state of increased hunger leads to greater responding to highly palatable food (Schéle et al., 2016; Bake et al., 2019). The present finding contradicts previous literature on voluntary abstinence, where rodents choose food over drug rewards (Caprioli et al., 2015; Venniro et al., 2016). It 's important to note that voluntary abstinence research has focused on operant conditioning, and the present study demonstrates that the incentive motivational properties of the cocaine-associated context are greater than those of the chocolate-reward context. This result aligns with previous findings that report a preference for the cocaine-associated cue over the sucrose-associated cue during cue-induced reinstatement in a mouse-contingent model of dual cocaine and sucrose SA (Bobadilla et al., 2020). In both contingent and noncontingent dual-reward paradigms, we conclude that the drug-associated cue or context may exert stronger conditioned contextual influence under these CPP conditions.

Regarding TRAP2-labeled neuronal populations tagged during exposure to reward-paired contexts, we observed a high tdTomato^+^ cell density in the PL in both single- and dual-condition CPP paradigms (Fig. 4*D*). This finding is consistent with previous literature (Cruz et al., 2014 ; Kane et al., 2021) showing that the PL has higher levels of c-Fos expression following reward-seeking behaviors. Although baseline c-Fos levels are low in both regions, the PL shows stronger cue- and context-evoked c-Fos modulation than the NAcore (Kim et al., 2015; Roy et al., 2022), potentially biasing the ensemble tagging mechanism via c-Fos-TRAP2. Together, these results suggest that the PL engages a high amount of activity-tagged ensembles during exposure to reward-paired contexts, likely reflecting region-specific differences in c-Fos-driven ensemble tagging (Goldstein and Volkow, 2002; Ikegami et al., 2007).

We also found that ensembles tagged during saline-, cocaine-, and chocolate-paired contexts were similar in size in the NAcore, regardless of whether single- or dual-reward paradigms were used. Comparable tdTomato^+^ cell density in the NAcore associated with different rewards has also been observed in a contingent model of cocaine and sucrose SA (Bobadilla et al., 2020). Surprisingly, although mice did not develop a preference for either saline-paired chamber (Extended Data Fig. 1-1*C*), we observed a saline-context ensemble in the NAcore that was comparable to the reward-associated ensembles (Fig. 4*C*). We propose that the saline-associated ensemble reflects experimental context, specifically the distinct CPP chambers. This interpretation is supported by the absence of tdTomato^+^ expression in the home cage control group (Fig. 4) and by the lack of correlation with locomotion on test day (Extended Data Fig. 1-4). This observation underscores the importance of context in both contingent and noncontingent reward models, as evidenced by the increase in tdTomato^+^ cell density during exposure to CPP chambers compared with a familiar home cage. By observing an increase in tdTomato^+^ cell density in both the context-only and context-with-rewards conditions, we believe that reward-paired ensembles reflect both contextual and reward-associated activity. Interestingly, while a saline-context ensemble was observed in the PL, it was larger than the home cage and cocaine 4-OHT^−^ groups yet smaller than the single-reward–exposed ensembles (Fig. 4*D*). This suggests that the density of TRAP2-labeled tdTomato^+^ cells may distinguish repeated exposure to rewarding versus nonrewarding contexts in the PL. This finding contradicts previous work that showed that acute exposure to cocaine increased c-Fos expression in the NAcore but not the PL (Nawarawong and Olsen, 2020). This may be because a single exposure to cocaine is insufficient to induce high levels of c-Fos. In contrast, repeated exposure to contexts or stimuli in the PL produces stronger activation (DeNardo et al., 2019).

We also examined whether biological sex influenced TRAP2-labeled neuronal populations. Although sex differences have been reported in cocaine-related behaviors and neural plasticity, we did not detect sex-dependent differences in tdTomato-labeled cells in either the NAcore or PL. Instead, differences in tdTomato^+^ cell density were driven by reward condition rather than sex. While the present study may not have been powered to detect subtle sex-dependent neural differences, these results suggest that the recruitment of context-activated neuronal populations in these regions during the CPP test was similar in male and female mice.

We employed c-Fos-TRAP2 transgenic mice (DeNardo et al., 2019) in our tagging method, which leverages c-Fos expression as a proxy for neuronal activation. It is essential to acknowledge the temporal limitations of this technique, as other methods have leveraged calcium-based activity–dependent tools with shorter tagging windows (Pang et al., 2024; Zhang et al., 2024). For instance, other techniques assess neuronal firing via calcium signaling in vivo to provide insights into neuronal activity-dependent population recruitment and specificity (Siciliano et al., 2019; Davidson et al., 2023). However, our findings on tdTomato^+^ cell density align with previous research across different drugs of abuse, underscoring the importance of c-Fos-expressing neuronal ensembles as markers of recent activity (Bossert et al., 2011; Fanous et al., 2012; Cruz et al., 2015; Rubio et al., 2019).

In this study, we did not find any correlation between tdTomato^+^ cell density and time spent in the cocaine-paired chamber (Extended Data Fig. 1-2) but did note a significant correlation between time spent in the chocolate-paired chamber and tdTomato^+^ cell density in the NAcore (Extended Data Fig. 2-1). This differs from previous results using contingent protocols, where significant correlations have been found between the size of c-Fos-TRAP-tagged ensembles in the NAcore and the level of cocaine-seeking behavior after single- and dual-reward SA, but not correlating with single sucrose-seeking behavior (Bobadilla et al., 2020). This contrasting finding highlights that the chosen behavioral model is essential for ensemble correlations and that noncontingent modeling differs from contingent SA models in this respect. We suggest that the lack of correlation suggests that ensemble size may play a more prominent role in contingent models, in which motivation and effort are required to obtain the reward, but not in noncontingent paradigms where access is guaranteed. This observation provides valuable insight into how behavioral context shapes neural coding of reward. Studies on ensembles using c-Fos-TRAP2 in fear recall suggest that the learning duration (DeNardo et al., 2019) or the level of activation of individual cells during memory recall (Leake et al., 2021; Hammack et al., 2023; Thibeault et al., 2024; Dovek et al., 2025) may be stronger indicators of the role of ensembles on operant behavior. A similar interpretation was proposed in recent work showing that repeated noncontingent cocaine exposure reduced ensemble size while increasing ensemble signaling strength (Thibeault et al., 2024). This suggests that ensemble synaptic connectivity may be more important than ensemble size. A limitation of CPP is that it requires animals to learn reward-associated behaviors with less effort than operant conditioning (McKendrick and Graziane, 2020). Although SA protocols often yield findings that overlap with CPP (Bardo and Bevins, 2000), motivational differences persist, underscoring the importance of both models in studying distinct phases of SUD (Green and Bardo, 2020). It is important to emphasize that only the groups that underwent the CPP protocols and received 4-OHT injections established TRAP2-labeled ensembles of neurons active during the test context and rewards-associated cues in the NAcore (Fig. 4*C*,*D*). Additionally, ensembles tagged during exposure to drug- and nondrug-paired contexts were significantly larger in the PL than those associated with context alone (saline CPP group) or home cage control groups (Fig. 4*C*,*D*). Thus, in this nonoperant model, PL tdTomato^+^ cell density varies across behavioral conditions, while the NAcore showed a consistent increase following CPP from the home cage.

### Conclusions

We developed a model of noncontingent dual cocaine–chocolate CPP, enabling us to characterize and compare drug- and nondrug-associated ensemble formation following polyreward exposure to single-reward exposure. Given the growing interest in ensemble-centric studies within SUD research, characterizing neuronal ensembles in preclinical models of polyreward exposure with strong face validity is crucial for advancing our understanding of SUD complexities and identifying potential therapeutic targets.

## Data Availability

The raw and analyzed data that support the findings of this study are available from the corresponding author upon reasonable request.

## Synthesis

Reviewing Editor: Sam Golden, The University of Washington Decisions are customarily a result of the Reviewing Editor and the peer reviewers coming together and discussing their recommendations until a consensus is reached. When revisions are invited, a fact-based synthesis statement explaining their decision and outlining what is needed to prepare a revision will be listed below. The following reviewer(s) agreed to reveal their identity: NONE.

Dear Bobadilla,

Your manuscript has been reviewed by two experts in the field, who both found the manuscript of high interest and technical merit. They have provided comments that will strengthen the manuscript, mostly relating to an improved categorization and discussion of the TRAP methods used. Please provide a point-by-point rebuttal and revised manuscript. I look forward to re-reviewing the revised work.

Reviewer 1:

The present paper focuses on developing a dual-reward CPP paradigm (cocaine vs. chocolate) in c-Fos-TRAP2 mice and using tdTomato labeling to tag CPP-related neuronal populations in PL and NAcore during a preference test. The overall goal is to compare drug versus natural reward ensembles and relate ensemble size to behavioral preference. While it is overall well written, several concerns limit my enthusiasm. Find them listed below on a point by point basis.

Major concerns

1) At present, the data support activity-dependent labeling, but not necessarily functional neuronal ensembles (coordinated, causally relevant cell assemblies). This is exacerbated by several findings. First, the find ensemble size does not correlate with reward-seeking across single or dual conditions. Second, saline labeling in NAcore is comparable to reward labeling, and PL saline labeling is not clearly separable from dual. Another issue with the dual labeling group is that dual CPP does not cleanly isolate cocaine- vs chocolate-seeking ensembles within the same subject. tdTomato labeling occurs after a 30-min test in which the animal traverses both contexts. As written, the manuscript treats this largely as a cocaine-seeking ensemble because of a modest preference, but the labeling window plausibly captures a mixture of exploration, context processing, and both cue sets. I suggest the authors tone down ensemble language throughout unless supported by additional causal role evidence

2) The discussion suggests translational implications by saying selective manipulation of drug ensembles could spare natural reward processes, but the current paper does not causally manipulate ensembles, nor demonstrate separable drug vs natural reward ensembles in a way that supports sparing.

3) The main behavioral result is a context preference for the cocaine side aafter dual reward training. The manuscript generalizes this in a way that reads as a broad statement about reward hierarchy, suggesting cocaine seeking overrides food seeking. However, the voluntary abstinence literature demonstrates that under many conditions rodents reliably choose food over drug and will maintain abstinence when food is available. The authors should narrow their claims in a revised discussion.

4) The manuscript repeatedly tests preference against 50% chance, including describing a "superiority design". In CPP, the more informative analysis is within-subject comparisons (reward vs vehicle chamber time, or cocaine vs chocolate chamber time in dual), and change scores from pre-test to test. I suggest making the paired comparisons the primary comparisons and demote one-sample vs 50% to a secondary check.

5) Locomotion is reported for conditioning sessions, but not for the test day. Given the relatively high saline-context labeling and the null correlations, test-day locomotion is a major potential confound that should be reported. I also suggest including a test to see whether test-day locomotion correlates with tdTomato expression.

6) The manuscript reports robust cocaine-day locomotion increases in the dual paradigm compared to cocaine

Reviewer 2:

Authors use the conditioned place preference model with either single (cocaine or chocolate) or dual rewards (cocaine and chocolate) to examine ensembles within the prefrontal cortex and the nucleus accumbens core using FosTRAP2 x Ai14 transgenic mice. They systematically compare male and female mice in their behavior, although this analysis is missing from their assessment of ensembles. They find similar ensemble sizes in both regions across reward types. It is an interesting question and approach, but some questions about data analysis and the behavioral methods and approach to cell quantification need to be clarified or redone prior to publication. See detailed comments below:

1. Authors state that they have found a "within subjects" approach to study cocaine and chocolate ensembles using FosTRAP2 x Ai14 transgenic mice. I understand that the authors are able to condition the mice for both drug and natural rewards, but none of the data provided here shows a within subjects comparison between the ensembles per se. In all cases throughout the manuscript, a single ensemble is tagged and examined, and the comparison is made between subjects. It would be possible to use a within subjects approach if authors labeled two separate ensembles within the same mice, for example by first tagging with 4-OHT and then by labeling a second ensemble using Fos immunohistochemistry (or some other activity-dependent labeling based approach) but as it stands, there is no within subjects examination of the cocaine and chocolate CPP ensembles.

2. It is unclear to me if the 3-7 hours of ad libitum food that the authors describe in the methods section is supposed to restrict their food intake or not. Given that their level of satiety will change their motivation to seek the food associated context this needs to be clarified. It would also be interesting to know if food restriction shifts their preference toward the chocolate-associated side of the chamber for the dual CPP procedure. It is worth noting more clearly that mice prefer the cocaine side even when food restricted if this is the case.

3. Authors use a 3-chamber apparatus but don't include if they exclude any animals based on time spent in the center chamber. Typically if mice spend a lot of time in the middle chamber (greater then 25-30% of the session) then this can inflate the percentage of CPP, and sometimes suggests that the mice are anxious or stressed, so it is necessary to exclude them on these grounds. It would be useful to re-examine the data to make sure that no mice need to be excluded on this basis. If authors have already done this, it should be stated in the methods.

4. Authors show data pertaining to locomotion and list "movement counts" in Figures 1-3 but there is no description of how this is done (beam breaks, video tracking, manual counting?) in the methods section, so this should be added prior to publication.

5. In Figure 1 it is helpful that the authors have included a timeline of the behavioral experiment, but it would be nice to also include when the tamoxifen injection is given relative to the final session. This tends to vary a lot in the literature so it would be informative to include.

6. In Figure 1C and D,Figure 2C and D, and Figure 3C and D the CPP data are presented in terms of percentage of time spent in the reward-paired chamber which is a useful way to present the data, but it would also be informative to see the data in terms of seconds spent in the chamber. Knowing the actual magnitude of the CPP in terms of seconds is a helpful way to assess the strength of the conditioning as well. This can be added to what is currently presented.

7. The authors also mention that they determine whether mice show CPP by comparing the percentage of time spent in the paired chamber with 50%. This does not give a particularly accurate measurement of the individual mouse's CPP score. A more accurate approach would be to compare the amount of time spent in the reward-paired chamber to the time spent in that chamber during the pretest (post test - pre test) or alternatively you can report the time spent in the reward-paired chamber relative to the unpaired chamber on test day (time in paired - time in unpaired). Please redo the analysis using either of these approaches to give a more accurate assessment of preference

8. Authors report that side assignment was counterbalanced for the dual conditioning procedure. Does this mean that side assignment was biased for the single reward conditioning experiments? If so can the authors explain why they chose to do this rather than counterbalancing for all experiments?

9. To the authors credit they present a clear analysis of males vs. females in all behavior graphs which is very interesting and informative. It would be nice to have some indication also in the overall preference graphs of which mice are male or female (Figures 1C, 2C, 3C). Maybe just by using a different dot shape to indicate male or female. Unfortunately, the male vs. female comparison disappears for the ensemble part of the paper (Fig 4 and 5). Why not also include the sex comparison for ensemble sizes in PLC and NAc? This would be a very informative addition to the paper and would increase the novelty of the findings.

10. I am a bit unclear on whether the data in Figure 4 are different from Figure 5 or not? It appears that authors have taken specific groups from the experiment in Figure 5 and highlighted them in Figure 4 and done a statistical comparison between the 2 brain regions without including the other control groups. If I am reading it correctly the same thing could have been done just by presenting Figure 5C and D on the same graph and running a direct comparison there. This would also be more statistically correct because it would use an ANOVA to compare all groups against their controls and in the event of a significant interaction, postdoc testing can be used to identify differences between groups without cherry picking groups to compare via t-test.

11. It is helpful that authors included an experiment in which 4-OHT is not on board to show that there is not non-specific expression of tdTomato in this model

12. Authors report the percentage of TRAPed cells relative to the NeuN-positive population in Figures 4 and 5. This is interesting and informative but it would also be interesting to see either raw cell counts or cell counts per unit of area to better understand the differences between groups. Since the number of NeuN expressing cells is orders of magnitude more than the TRAPed cells, even small variations in NeuN count can shift the results when reporting percentages. Currently, it looks like there are very low percentages overall which is good to know because we know that ensembles are a very small percentage of the neuronal population. But in addition it would be very useful to know the counts for tdTomato in all groups and the counts for NeuN. I appreciate the inclusion of example images for all groups, this is very helpful.

## Author Response

Dear editor and reviewers, We thank the reviewers for their time and effort dedicated to evaluating our manuscript, entitled "Characterization of neuronal ensembles in a model of dual reward conditioned place preference." We appreciate the constructive feedback, which has substantially improved the clarity and rigor of our manuscript. In response to these comments, we have made significant revisions throughout the text, figures, and analysis.

Importantly, these revisions strengthen the main takeaway of this study: The development of a novel dual-reward paradigm using cocaine and chocolate in alternating conditioning sessions in tandem with activity-based tagging using the TRAP2 system. This provides a robust framework for examining TRAP2-labeled cell expression by competing reward contexts.

Below, we provide a detailed point-by-point response to each reviewer in blue, italicized text. All revisions have been clearly indicated with the same blue text in the manuscript. However, because reviewers raised overlapping concerns, we first address these major issues collectively before presenting our individual responses.

Major Concern #1: Revision of the CPP test day data to raw scores instead of percentages Reviewers highlighted the limitations of percentage-based analysis and suggested reporting raw time. In response, we have revised all CPP test-day analyses to use the raw time spent in each conditioned chamber. These changes can be seen in all behavioral figures (figs. 1C, E, 2C, E, 3C, E, 1-1C, E and 1-2C, E), to improve transparency of the CPP test day behavior.

Major Concern #2: Revision of the CPP test day analysis to prioritize within-subject comparisons Reviewers encouraged us to improve the statistical rigor of our manuscript. To accomplish this, we adjusted our statistical analysis of test-day raw time to within-subject paired comparisons (reward vs. vehicle, or cocaine vs. chocolate in the dual paradigm on test day) as the primary statistical test. This was done to prevent statistical bias from using a biased design in single-reward CPP (preconditioning the least-preferred chamber as the reward chamber) and a counterbalanced design in dual-reward CPP (random assignment of reward chambers). Importantly, this updated analytical approach did not alter the overall pattern of results or the study's conclusions.

Your manuscript has been reviewed by two experts in the field, who both found the manuscript of high interest and technical merit. They have provided comments that will strengthen the manuscript, mostly relating to an improved categorization and discussion of the TRAP methods used. Please provide a point-by-point rebuttal and revised manuscript. I look forward to re-reviewing the revised work.

Reviewer 1:

The present paper focuses on developing a dual-reward CPP paradigm (cocaine vs. chocolate) in c-Fos-TRAP2 mice and using tdTomato labeling to tag CPP-related neuronal populations in PL and NAcore during a preference test. The overall goal is to compare drug versus natural reward ensembles and relate ensemble size to behavioral preference. While it is overall well written, several concerns limit my enthusiasm. Find them listed below on a point by point basis.

Major concerns 1) At present, the data support activity-dependent labeling, but not necessarily functional neuronal ensembles (coordinated, causally relevant cell assemblies). This is exacerbated by several findings. First, the find ensemble size does not correlate with reward-seeking across single or dual conditions. Second, saline labeling in NAcore is comparable to reward labeling, and PL saline labeling is not clearly separable from dual. Another issue with the dual labeling group is that dual CPP does not cleanly isolate cocaine- vs chocolate-seeking ensembles within the same subject. tdTomato labeling occurs after a 30-min test in which the animal traverses both contexts. As written, the manuscript treats this largely as a cocaine-seeking ensemble because of a modest preference, but the labeling window plausibly captures a mixture of exploration, context processing, and both cue sets. I suggest the authors tone down ensemble language throughout unless supported by additional causal role evidence.

We agree with the reviewers and have revised the manuscript throughout to clarify that TRAP2-labeled neurons represent activity-dependent-tagged populations, not causally verified ensembles. We explicitly state that the tagged population reflects activity across both contexts during the 30-minute test session in dual. We removed or softened all causal language throughout.

2) The discussion suggests translational implications by saying selective manipulation of drug ensembles could spare natural reward processes, but the current paper does not causally manipulate ensembles, nor demonstrate separable drug vs natural reward ensembles in a way that supports sparing.

We have removed speculative translational statements and now explicitly state that the present study does not manipulate ensembles and therefore cannot infer causal roles. The revised Discussion emphasizes that our findings are descriptive and do not support claims about selective sparing.

3) The main behavioral result is a context preference for the cocaine side aafter dual reward training. The manuscript generalizes this in a way that reads as a broad statement about reward hierarchy, suggesting cocaine seeking overrides food seeking. However, the voluntary abstinence literature demonstrates that under many conditions rodents reliably choose food over drug and will maintain abstinence when food is available. The authors should narrow their claims in a revised discussion.

We have substantially narrowed our claims in the resubmission version of the manuscript. The Discussion now states that our findings reflect contextual preference in a non-contingent CPP paradigm rather than a general hierarchy of reward value. We added citations contrasting CPP with voluntary abstinence and operant choice paradigms (Caprioli et al., 2015; Venniro et al., 2016) and clarified that our results apply only to context-driven preference. We also explicitly note that mice preferred the cocaine-paired context even under food-restricted conditions, but we avoid interpreting this as a universal dominance of cocaine reward.

4) The manuscript repeatedly tests preference against 50% chance, including describing a "superiority design". In CPP, the more informative analysis is within-subject comparisons (reward vs vehicle chamber time, or cocaine vs chocolate chamber time in dual), and change scores from pre-test to test. I suggest making the paired comparisons the primary comparisons and demote one-sample vs 50% to a secondary check.

Please see the response to Major Concern #2 5) Locomotion is reported for conditioning sessions, but not for the test day. Given the relatively high saline-context labeling and the null correlations, test-day locomotion is a major potential confound that should be reported. I also suggest including a test to see whether test-day locomotion correlates with tdTomato expression.

We have included test-day locomotion for all CPP experiments (Figures 1D, 2D, 3D, 1-1D, and 1-4D). We also included the locomotion-tdTomato+ cell density correlation in saline CPP animals and report that locomotion does not correlate with tdTomato expression (Figure 1-3).

6) The manuscript reports robust cocaine-day locomotion increases in the dual paradigm compared to cocaine We corrected this typo to accurately describe locomotion in the dual paradigm during conditioning.

Reviewer 2:

Authors use the conditioned place preference model with either single (cocaine or chocolate) or dual rewards (cocaine and chocolate) to examine ensembles within the prefrontal cortex and the nucleus accumbens core using FosTRAP2 x Ai14 transgenic mice. They systematically compare male and female mice in their behavior, although this analysis is missing from their assessment of ensembles. They find similar ensemble sizes in both regions across reward types. It is an interesting question and approach, but some questions about data analysis and the behavioral methods and approach to cell quantification need to be clarified or redone prior to publication. See detailed comments below:

1. Authors state that they have found a "within subjects" approach to study cocaine and chocolate ensembles using FosTRAP2 x Ai14 transgenic mice. I understand that the authors are able to condition the mice for both drug and natural rewards, but none of the data provided here shows a within subjects comparison between the ensembles per se. In all cases throughout the manuscript, a single ensemble is tagged and examined, and the comparison is made between subjects. It would be possible to use a within subjects approach if authors labeled two separate ensembles within the same mice, for example by first tagging with 4-OHT and then by labeling a second ensemble using Fos immunohistochemistry (or some other activity-dependent labeling based approach) but as it stands, there is no within subjects examination of the cocaine and chocolate CPP ensembles.

We agree with the reviewer and removed language implying a within-subject ensemble comparison. We now state more clearly that ensembles were compared between subjects, and that dual CPP tags a mixed population of TRAP2-labeled neurons from both contexts during test day.

2. It is unclear to me if the 3-7 hours of ad libitum food that the authors describe in the methods section is supposed to restrict their food intake or not. Given that their level of satiety will change their motivation to seek the food associated context this needs to be clarified. It would also be interesting to know if food restriction shifts their preference toward the chocolate-associated side of the chamber for the dual CPP procedure. It is worth noting more clearly that mice prefer the cocaine side even when food restricted if this is the case.

We clarified the food-access schedule in the methods and explained that the restricted window was intended to ensure chocolate consumption during conditioning. We now explicitly note that mice still preferred the cocaine-paired chamber, despite being food-restricted. We address this point in the revised Discussion.

3. Authors use a 3-chamber apparatus but don't include if they exclude any animals based on time spent in the center chamber. Typically if mice spend a lot of time in the middle chamber (greater then 25-30% of the session) then this can inflate the percentage of CPP, and sometimes suggests that the mice are anxious or stressed, so it is necessary to exclude them on these grounds. It would be useful to re-examine the data to make sure that no mice need to be excluded on this basis. If authors have already done this, it should be stated in the methods.

The reviewer raises an interesting point. We added a statement to the method describing our exclusion criterion as {greater than or equal to}40% of the time in the gray chamber. We based this on Hnasko et al (2007) and reported that, with this criterion, only one saline animal was excluded.

4. Authors show data pertaining to locomotion and list "movement counts" in Figures 1-3 but there is no description of how this is done (beam breaks, video tracking, manual counting?) in the methods section, so this should be added prior to publication.

We added a detailed description of the Med Associates infrared beam-break locomotion metric to the CPP methods section.

5. In Figure 1 it is helpful that the authors have included a timeline of the behavioral experiment, but it would be nice to also include when the tamoxifen injection is given relative to the final session. This tends to vary a lot in the literature so it would be informative to include.

We added explicit timing immediately after the test session, in both the methods and the Figure timelines (Figures 1, 2, 3, 1-1, and 1-4).

6. In Figure 1C and D, Figure 2C and D, and Figure 3C and D the CPP data are presented in terms of percentage of time spent in the reward-paired chamber which is a useful way to present the data, but it would also be informative to see the data in terms of seconds spent in the chamber. Knowing the actual magnitude of the CPP in terms of seconds is a helpful way to assess the strength of the conditioning as well. This can be added to what is currently presented.

Please see the response to Major Concern #1 7. The authors also mention that they determine whether mice show CPP by comparing the percentage of time spent in the paired chamber with 50%. This does not give a particularly accurate measurement of the individual mouse's CPP score. A more accurate approach would be to compare the amount of time spent in the reward-paired chamber to the time spent in that chamber during the pretest (post test - pre test) or alternatively you can report the time spent in the reward-paired chamber relative to the unpaired chamber on test day (time in paired - time in unpaired). Please redo the analysis using either of these approaches to give a more accurate assessment of preference Please see the response to Major Concerns #1 and #2.

8. Authors report that side assignment was counterbalanced for the dual conditioning procedure. Does this mean that side assignment was biased for the single reward conditioning experiments? If so can the authors explain why they chose to do this rather than counterbalancing for all experiments? We added a clear justification: biased assignment is used in single-reward CPP to test whether conditioning can shift preference, whereas dual CPP requires counterbalancing to avoid systematic pairing of one reward with the preferred side.

9. To the authors credit they present a clear analysis of males vs. females in all behavior graphs which is very interesting and informative. It would be nice to have some indication also in the overall preference graphs of which mice are male or female (Figures 1C, 2C, 3C). Maybe just by using a different dot shape to indicate male or female. Unfortunately, the male vs. female comparison disappears for the ensemble part of the paper (Fig 4 and 5). Why not also include the sex comparison for ensemble sizes in PLC and NAc? This would be a very informative addition to the paper and would increase the novelty of the findings.

We included a two-way ANOVA comparing tdTomato expression by sex for both the NAcore and PL (Figure 5). No sex effects or interactions were detected on tdTomato expression.

10. I am a bit unclear on whether the data in Figure 4 are different from Figure 5 or not? It appears that authors have taken specific groups from the experiment in Figure 5 and highlighted them in Figure 4 and done a statistical comparison between the 2 brain regions without including the other control groups. If I am reading it correctly the same thing could have been done just by presenting Figure 5C and D on the same graph and running a direct comparison there. This would also be more statistically correct because it would use an ANOVA to compare all groups against their controls and in the event of a significant interaction, postdoc testing can be used to identify differences between groups without cherry picking groups to compare via t-test.

We removed pairwise comparisons of PL and NAcore expression. We also revised the text to clarify that Figure 4 represents a group-level comparison, while Figure 5 includes sex as an additional factor. We also ensured that statistical analyses are consistent and not duplicative.

11. It is helpful that authors included an experiment in which 4-OHT is not on board to show that there is not non-specific expression of tdTomato in this model Thank you.

12. Authors report the percentage of TRAPed cells relative to the NeuN-positive population in Figures 4 and 5. This is interesting and informative but it would also be interesting to see either raw cell counts or cell counts per unit of area to better understand the differences between groups. Since the number of NeuN expressing cells is orders of magnitude more than the TRAPed cells, even small variations in NeuN count can shift the results when reporting percentages. Currently, it looks like there are very low percentages overall which is good to know because we know that ensembles are a very small percentage of the neuronal population. But in addition it would be very useful to know the counts for tdTomato in all groups and the counts for NeuN. I appreciate the inclusion of example images for all groups, this is very helpful.

We now report tdTomato+ cell density (cells/mm2) instead of percent over NeuN. We also added an analysis of NeuN cell density to the supplemental figures, indicating that some of our previous findings may have been confounded by NeuN expression.
