# Supplementary figures and images for: Characterization of Neuronal Ensembles in a Model of Dual-Reward Conditioned Place Preference
Source: eNeuro. 2026 Jun 24;13(6):ENEURO.0463-25.2026. doi: 10.1523/ENEURO.0463-25.2026 (PMC13312911; doi:10.1523/ENEURO.0463-25.2026)

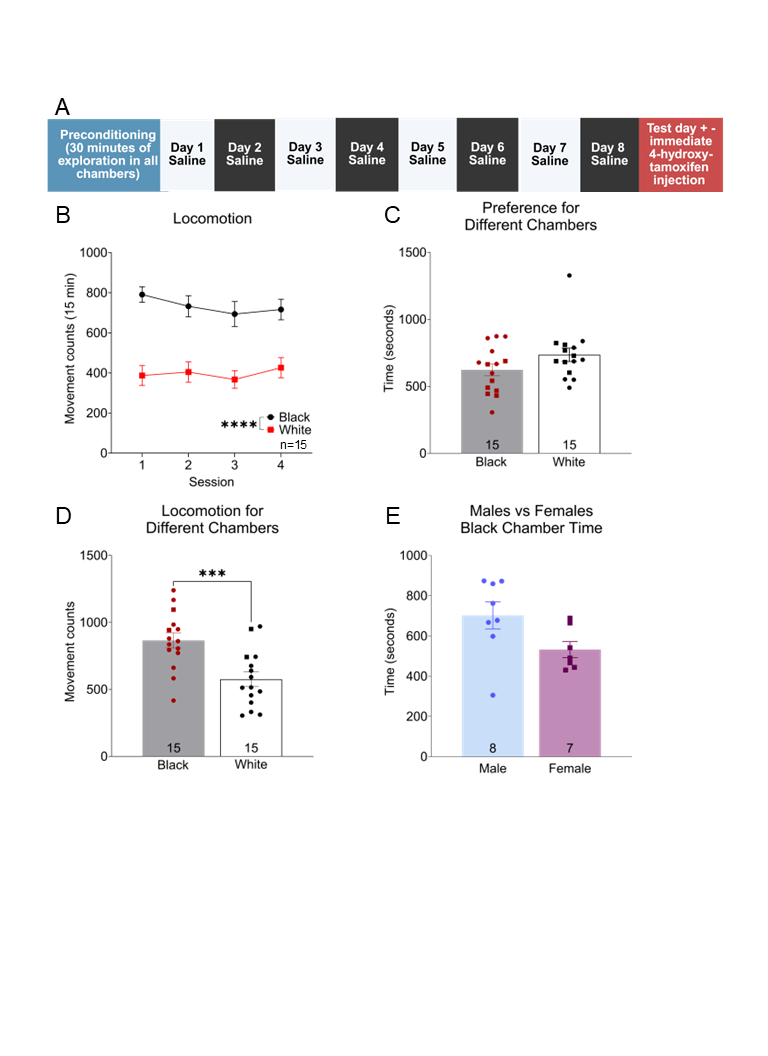

Supplement: Figure 1-1 — Saline CPP behavior. (A) Saline CPP timeline. Different colors denote different contexts. (B) Movement counts during both black and white conditioning sessions. **** p < 0.0001 Comparing black and white chamber movement conditioning. (C) Raw time during test day for the different contexts, paired t-test. (D) Locomotion of both chambers during test day, *** p < 0.001, paired t-test. (E) Sex differences in context preference were not observed, unpaired t-test. Numbers at the bottom of the bars indicate the sample size. Square data points represent females; circle data points represent males. Download Figure 1-1, TIF file. [file eneuro-13-ENEURO.0463-25.2026-s001.tif]

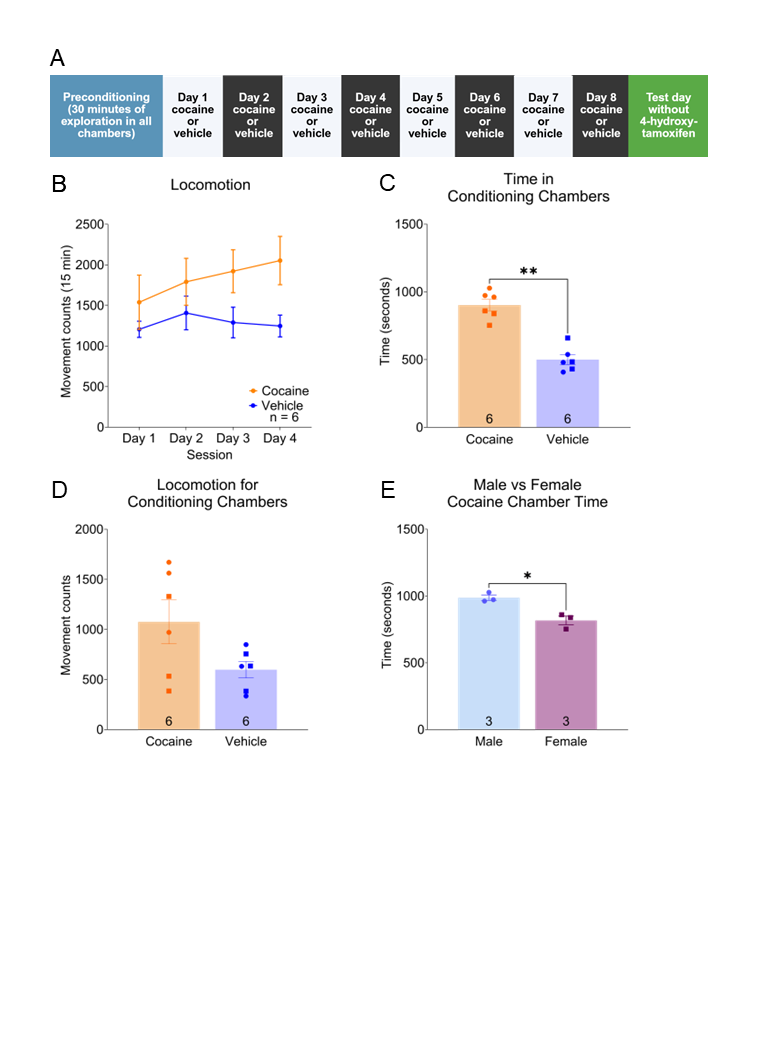

Supplement: Figure 1-2 — Cocaine CPP without 4-hydroxy-tamoxifen (4-OHT) behavior. (A) Cocaine CPP 4-OHT- timeline. Different colors denote different contexts. (B) Movement counts during conditioning sessions for the cocaine-paired chamber and the vehicle-saline chamber. (C) Raw time in each chamber on test day. ** p < 0.01 Comparing time in the cocaine-paired chamber to the vehicle-saline chamber, paired t-test. (D) Locomotion of both chambers during test day, paired t-test. (E) Males and females differ in time spent in the cocaine-paired chamber * p < 0.05, unpaired t-test. Numbers at the bottom of the bars indicate the sample size. Square data points represent females; circle data points represent males. Download Figure 1-2, TIF file. [file eneuro-13-ENEURO.0463-25.2026-s002.tif]

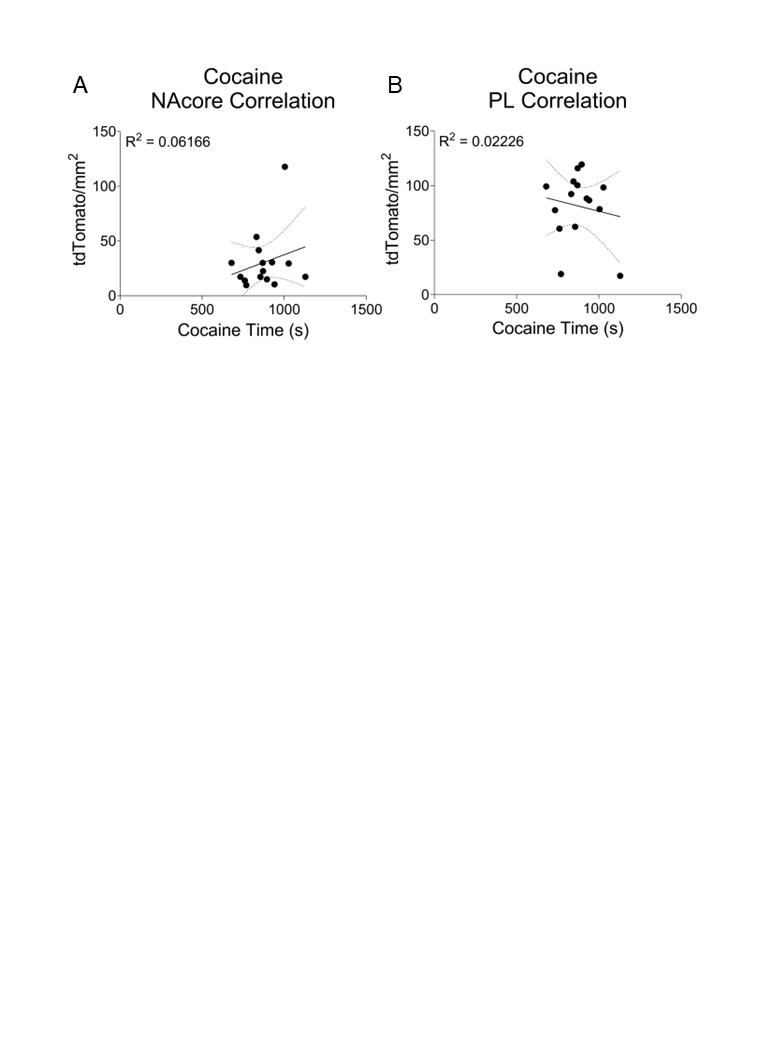

Supplement: Figure 1-3 — Correlations between cocaine-chamber time and tdTomato + cell density. (A) Correlation of cocaine-chamber time with the nucleus accumbens core (NAcore) tdTomato + cell density. (B) Correlation of cocaine-chamber time with the prelimbic cortex (PL) tdTomato + cell density. Download Figure 1-3, TIF file. [file eneuro-13-ENEURO.0463-25.2026-s003.tif]

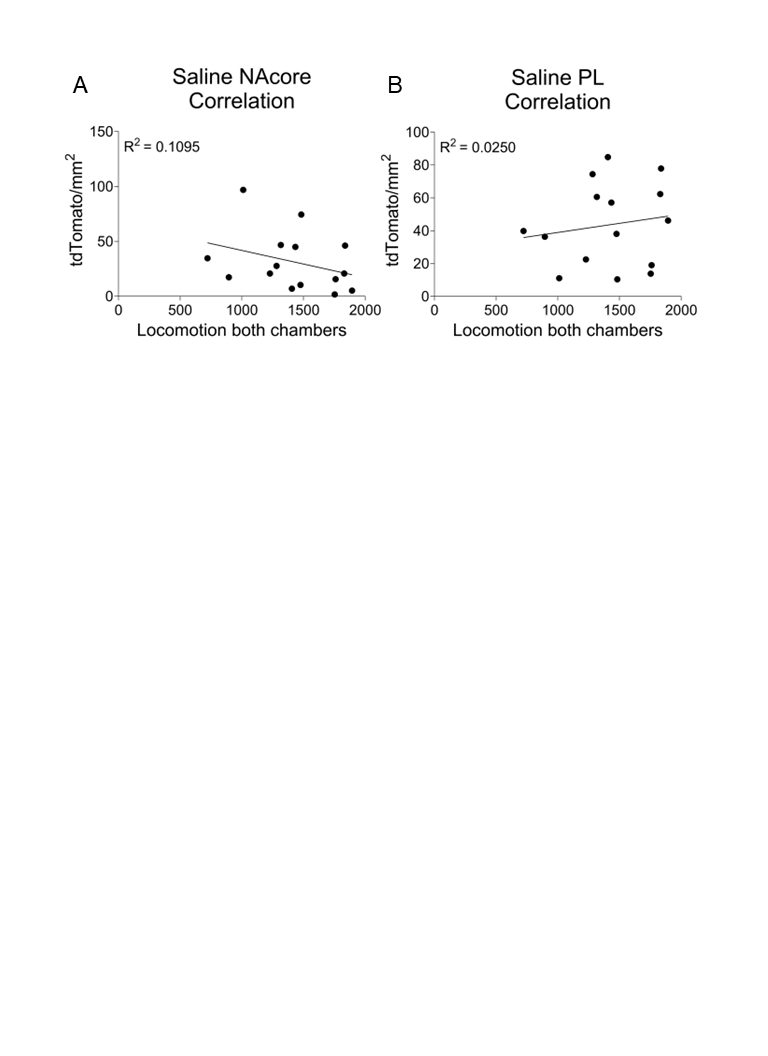

Supplement: Figure 1-4 — Correlations between saline locomotion and tdTomato + cell density. (A) Correlation of locomotion with the nucleus accumbens core (NAcore) tdTomato + cell density. (B) Correlation of locomotion with the prelimbic cortex (PL) tdTomato + cell density. Download Figure 1-4, TIF file. [file eneuro-13-ENEURO.0463-25.2026-s004.tif]

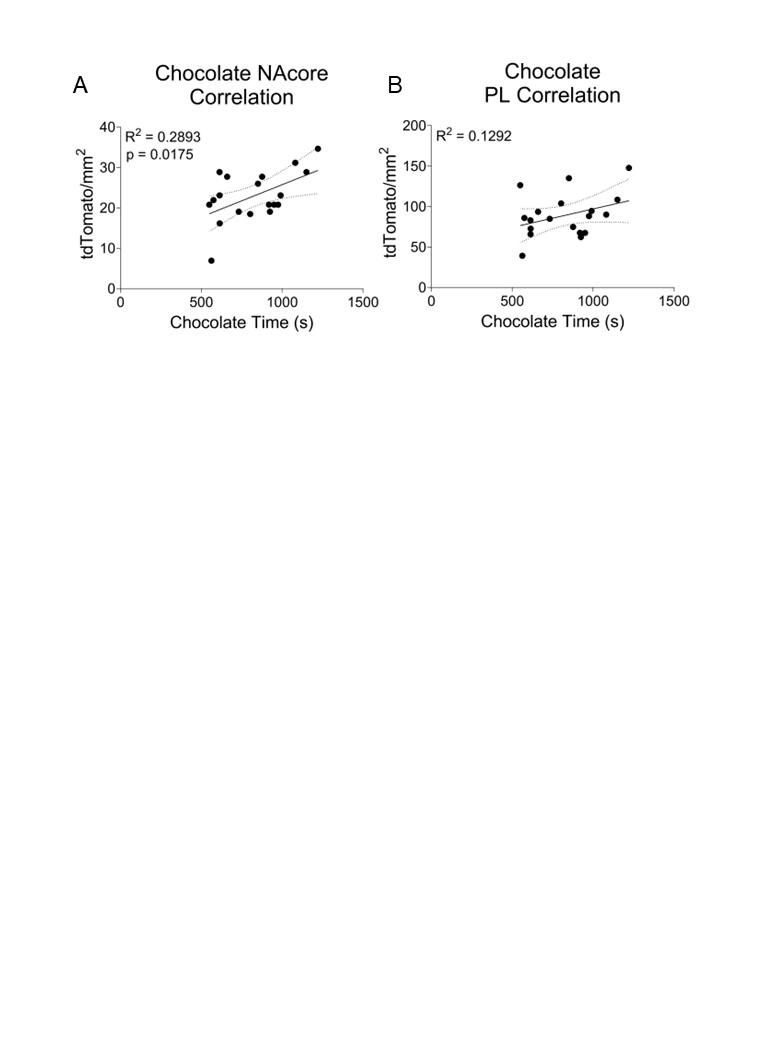

Supplement: Figure 2-1 — Correlations between chocolate-chamber time and tdTomato + cell density (A) Correlation of chocolate-chamber time with the nucleus accumbens core (NAcore) tdTomato + cell density. (B) Correlation of chocolate-chamber time with the prelimbic (PL) tdTomato + cell density. Download Figure 2-1, TIF file. [file eneuro-13-ENEURO.0463-25.2026-s005.tif]

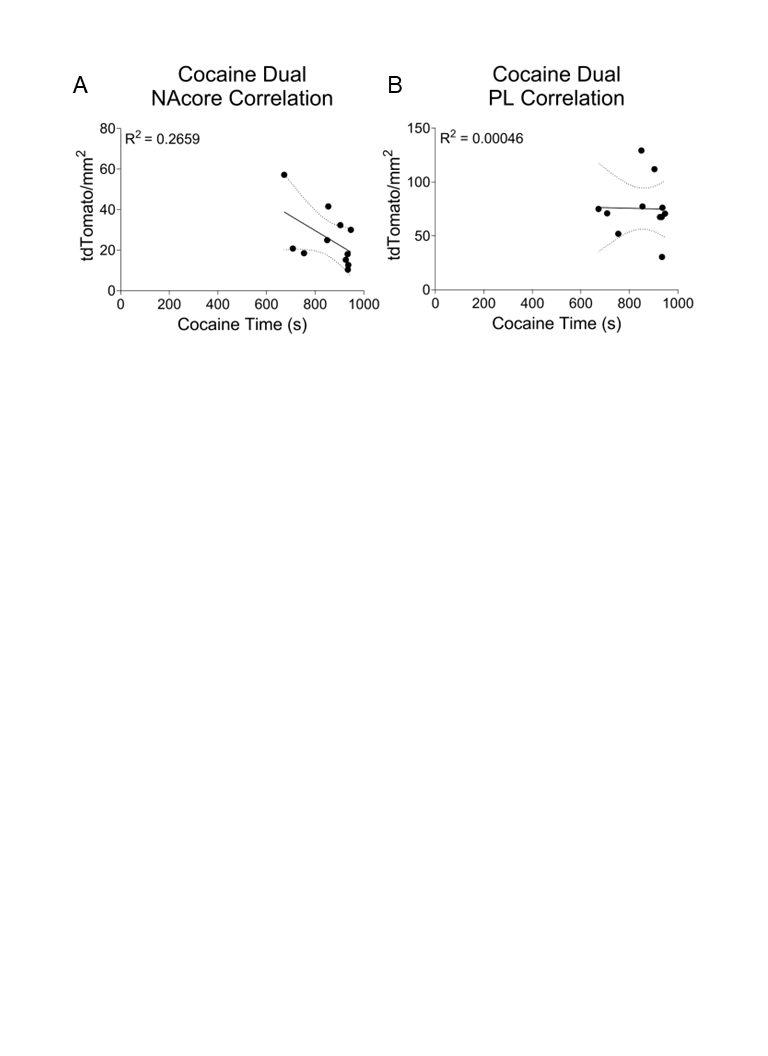

Supplement: Figure 3-1 — Correlation between cocaine-chamber time in dual conditioning with tdTomato + cell density. (A) Correlation of cocaine-chamber time in dual conditioning with nucleus accumbens core (NAcore) tdTomato + cell density. (B) Correlation of cocaine-chamber time in dual conditioning with the prelimbic cortex (PL) tdTomato + cell density. Download Figure 3-1, TIF file. [file eneuro-13-ENEURO.0463-25.2026-s006.tif]

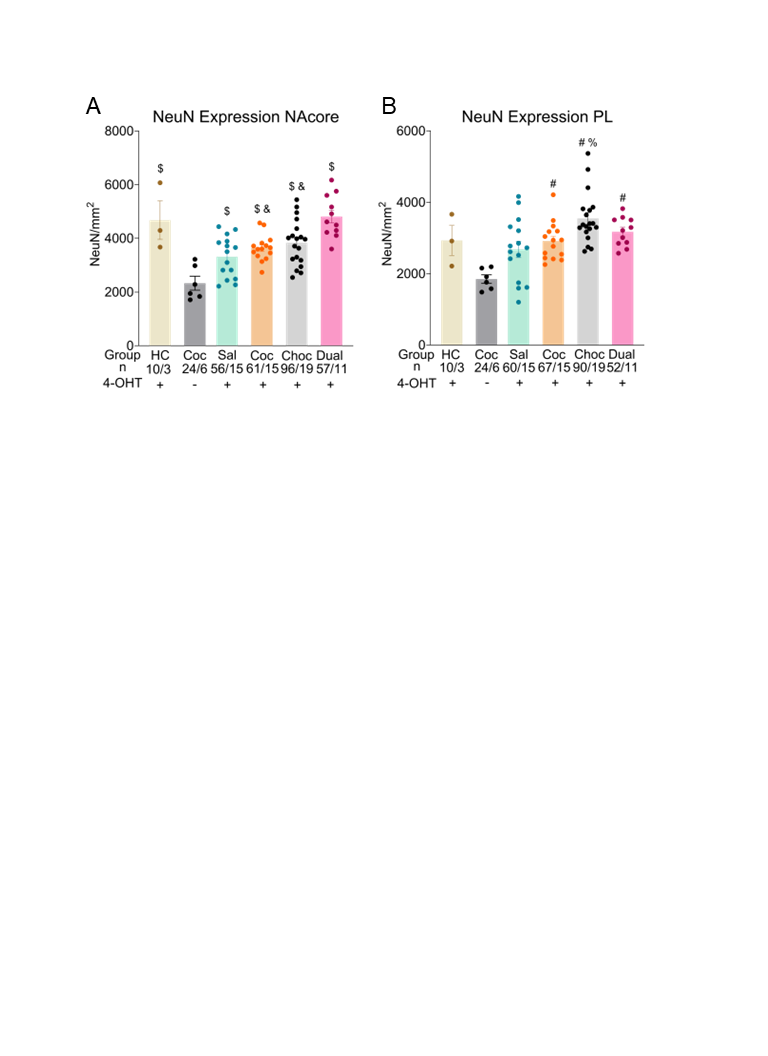

Supplement: Figure 4-1 — NeuN density across groups. (A) Expression of NeuN in the nucleus accumbens core (NAcore) differed across groups: home cage (HC), cocaine (Coc), saline (Sal), chocolate (Choc), cocaine and chocolate (dual), and 4-hydroxy-tamoxifen (4-OHT). $ p < 0.01 compared to cocaine 4-OHT-, & p < 0.05 compared to dual. (B) NeuN expression in the prelimbic cortex (PL) differed across groups. # p < 0.01 compared to saline, $ p < 0.05 compared to cocaine 4-OHT-. Numbers indicate images over behavioral sample size. Download Figure 4-1, TIF file. [file eneuro-13-ENEURO.0463-25.2026-s007.tif]

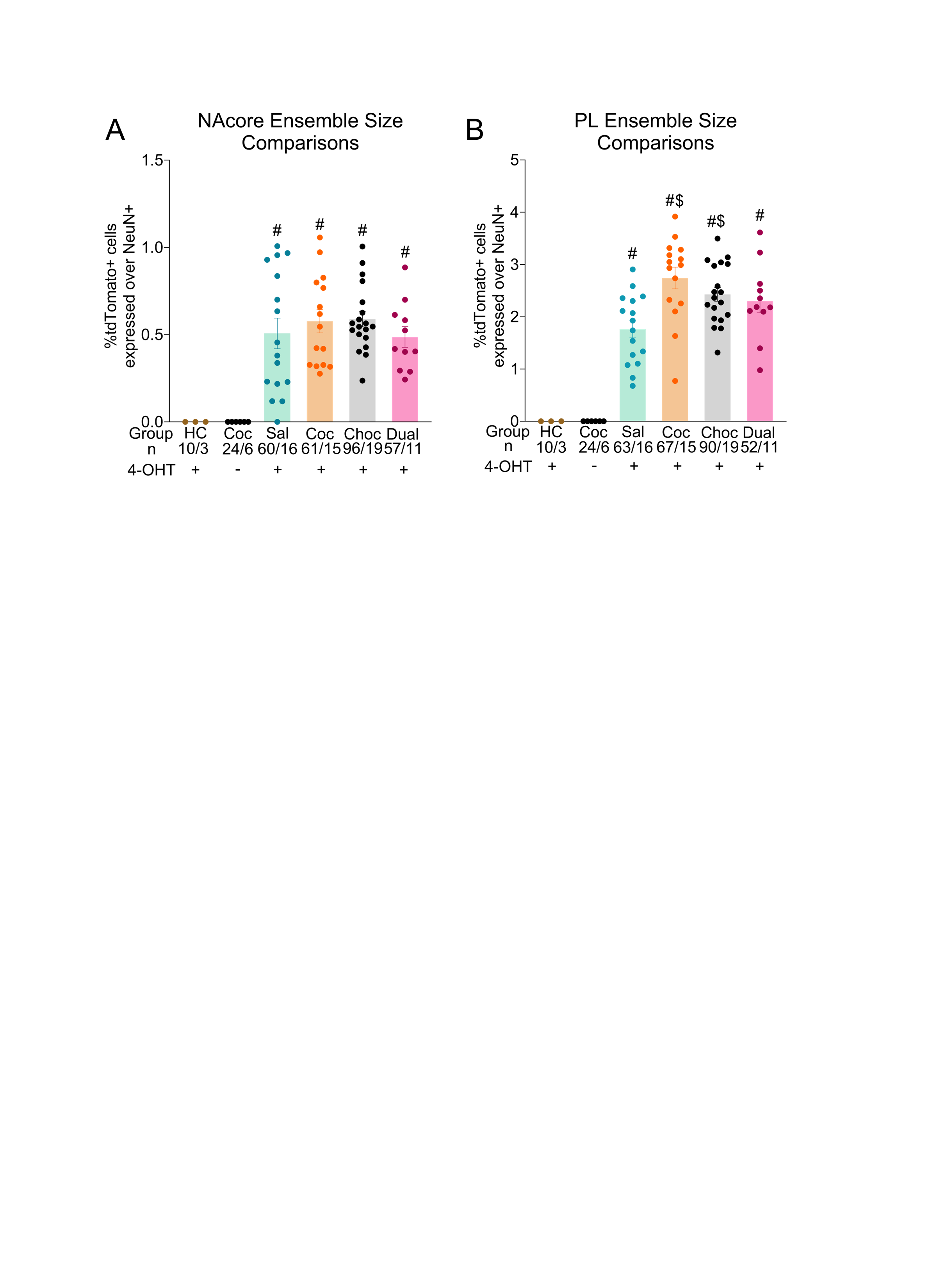

Supplement: Figure 4-2 — tdTomato expression normalized to NeuN. (A) Percent tdTomato+/ NeuN + cells in the nucleus accumbens core, home cage (HC), cocaine (Coc), chocolate (Choc), cocaine and chocolate (dual), and 4-hydroxytamoxifen (4-OHT). # p < 0.0001 compared to HC. (B) Percent tdTomato+/ NeuN + cells in the PL across reward groups. $ p < 0.0001 compared to saline. Numbers indicate the number of image acquisitions followed by behavioral sample size. Download Figure 4-2, TIF file. [file eneuro-13-ENEURO.0463-25.2026-s009.tif]
